# A long-lived magma ocean on a young Moon

**DOI:** 10.1126/sciadv.aba8949

**Published:** 2020-07-10

**Authors:** M. Maurice, N. Tosi, S. Schwinger, D. Breuer, T. Kleine

**Affiliations:** 1 German Aerospace Center (DLR), Rutherfordstr. 2, 12489 Berlin, Germany.; 2 Department of Astronomy and Astrophysics, Technische Universität Berlin, Berlin, Germany.; 3 Institut für Planetologie, University of Münster, Wilhelm-Klemm-Str. 10, 48149 Münster, Germany.

## Abstract

A giant impact onto Earth led to the formation of the Moon, resulted in a lunar magma ocean (LMO), and initiated the last event of core segregation on Earth. However, the timing and temporal link of these events remain uncertain. Here, we demonstrate that the low thermal conductivity of the lunar crust combined with heat extraction by partial melting of deep cumulates undergoing convection results in an LMO solidification time scale of 150 to 200 million years. Combining this result with a crystallization model of the LMO and with the ages and isotopic compositions of lunar samples indicates that the Moon formed 4.425 ± 0.025 billion years ago. This age is in remarkable agreement with the U-Pb age of Earth, demonstrating that the U-Pb age dates the final segregation of Earth’s core.

## Teaser

Fitting the isotopic composition of lunar rocks to a new thermal and crystallization model shows the Moon formed 4.40 to 4.45 Ga.

## INTRODUCTION

Most lunar formation models predict that the early Moon had a global magma ocean (*1*), which initially solidified from the bottom up via efficient radiative cooling to space at its hot liquid surface (*2*) or through a cold but very thin quenched crust (*3*). However, once buoyant plagioclase started to crystallize, it floated upward and formed an insulating lid (*4*), which markedly slowed down the cooling of the lunar magma ocean (LMO). As a result, the last ~20% of the LMO is thought to have crystallized on a longer time scale of 10 million years (Ma) (*2*) to 30 Ma (*3*). It has been proposed that tidal dissipation in the crust (*5*, *6*) or the magma ocean itself (*7*) may prolong the duration of the LMO solidification. Although it was initially proposed that this may result in an extended LMO lifetime of more than 200 Ma (*5*), a subsequent study by the same authors (*6*) has pointed out an error in the original model, which leads to a substantial overestimation of the LMO solidification time. Moreover, tidal dissipation mainly heats the uppermost layers of the crust and affects the magma ocean itself only on a reduced time scale (~10^4^ years). Thus, tidal dissipation does not seem to have had a strong influence on the LMO’s lifetime. Last, impacts of leftover debris from the Moon-forming event onto the lunar crust may have influenced the duration of LMO crystallization in two ways (*3*): Holes punctured in the crust may have accelerated cooling, and deposition of the impactors’ kinetic energy may have slowed it down. The net result of these two opposing effects is not well constrained, but the most recent results indicate that holes in the crust are short-lived and, therefore, did not significantly enhance the cooling of the LMO (*8*).

**Fig. 1:**
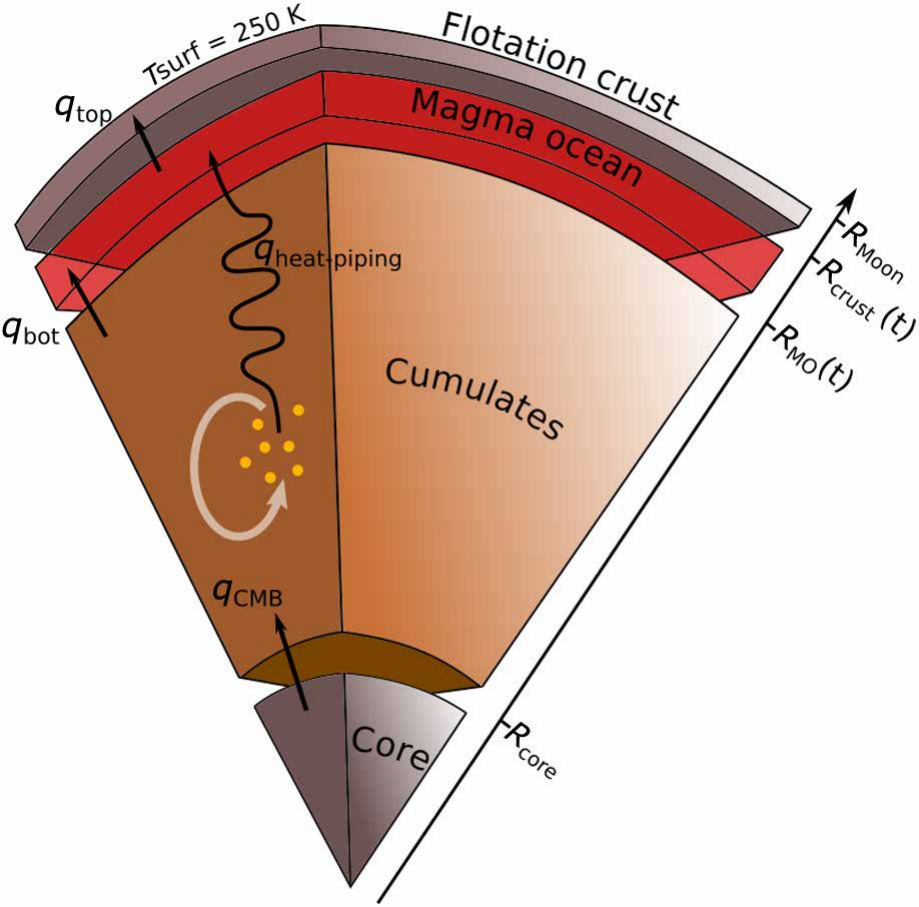
Sketch of the thermal evolution model with its four reservoirs. Black arrows indicate heat exchanges between different reservoirs. The gray arrow symbolizes solid-state convection in the cumulates, causing decompression melting (orange dots), melt migration, and heat piping. The radii of the interfaces between the reservoirs are represented on the inclined right axis.

The thermal conductivity (*k*_crust_) of the lunar crust, which determines the heat diffusion through it, is the key parameter controlling the influence of the crust on the LMO cooling time scale. The lunar crust is mainly composed of almost pure anorthite (*9*), a mineral having a very low thermal conductivity [~1.5 W m^−1^ K^−1^ (*10*)]. However, previous studies of LMO solidification (*2*, *3*, *5*) all used a much higher *k*_crust_ value of ~3.4 W m^−1^ K^−1^ and, therefore, likely underestimated the insulating efficiency of the crust. We tested the influence of *k*_crust_ on the LMO evolution using a thermal model containing four coupled reservoirs: core, solid cumulates, magma ocean, and flotation crust (Fig. 1). The thermal evolution is controlled by heat conduction or convection through the solid cumulates, conduction through the crust, and internal heating of the magma ocean. We initially set the temperature of the cumulates at their crystallization temperature, which we obtained, together with the internal heat production, from a crystallization model of initially 1350- (whole mantle), 1000-, 700-, and 500-km-deep magma oceans (see Materials and Methods). In a first series of simulations, we modeled the LMO solidification of a 1000-km-deep LMO [similar to (*2*)], assuming that the thermal evolution of the solid cumulates is solely governed by heat diffusion, while in a second series, we also considered the effects of cumulates undergoing solid-state convection. Last, we combined the time series of LMO solidification from the thermal model with a crystallization and chemical fractionation model. This combined model allows us to calculate the ^176^Lu-^176^Hf and ^147^Sm-^143^Nd isotopic evolution of the LMO, which can be fitted to the ages and isotopic compositions of lunar rocks and ultimately provides the onset time of LMO crystallization and, hence, the age of the Moon.

## RESULTS

**Fig. 2:**
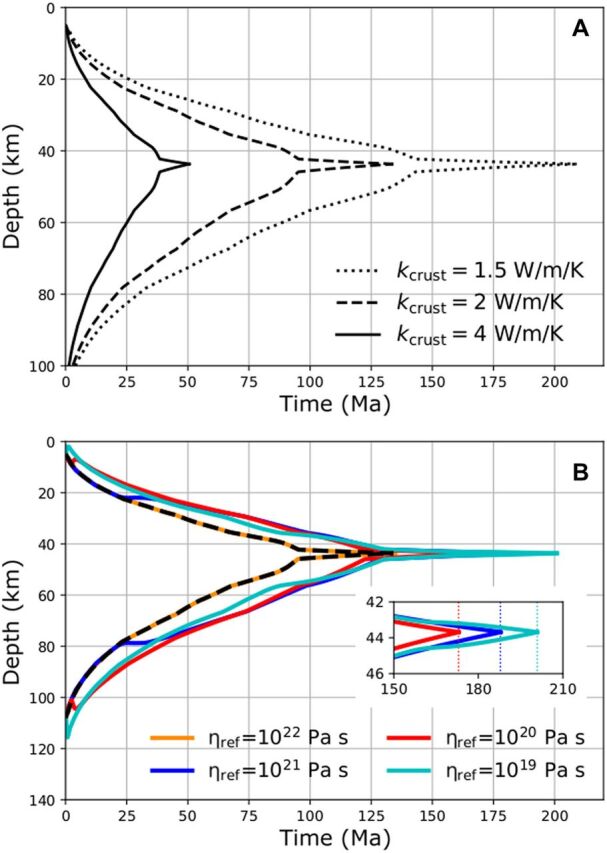
Time evolution of the LMO and crust’s bottom depth. (**A**) Evolution of the bottom radii of the LMO (lower curves) and of the crust (upper curves) for different values of the crustal thermal conductivity *k*_crust_, assuming purely conductive heat transfer through both the crust and the cumulates underlying the magma ocean. The protracted tail of solidification is due to the strong final drop in the crystallization temperature (see Materials and Methods). (**B**) As in (A) but for *k*_crust_ = 2 W m^−1^ K^−1^, taking into account thermal convection in the cumulates and the heat piping effect for different values of the reference viscosity of the cumulates η_ref_. The black dashed line corresponds to the same case on both panels. In the inset in (B), the vertical dotted lines in the inset indicate the time at which crystallization ends for different reference viscosities.

We modeled the evolution of the depth of the crystallization front (i.e., the bottom of the magma ocean) and of the bottom of the crust (Fig. 2A) for *k*_crust_ = 4, 2, and 1.5 W m^−1^ K^−1^. For *k*_crust_ = 4 W m^−1^ K^−1^ (used in previous models), complete solidification of the LMO takes 49 Ma, but when *k*_crust_ = 1.5 W m^−1^ K^−1^, the value of pure anorthosite, it takes up to 178 Ma. Because the lunar crust composition is not pure anorthosite, we take the slightly higher and conservative value of *k*_crust_ = 2 W m^−1^ K^−1^ for our fiducial case.

**Fig. 3:**
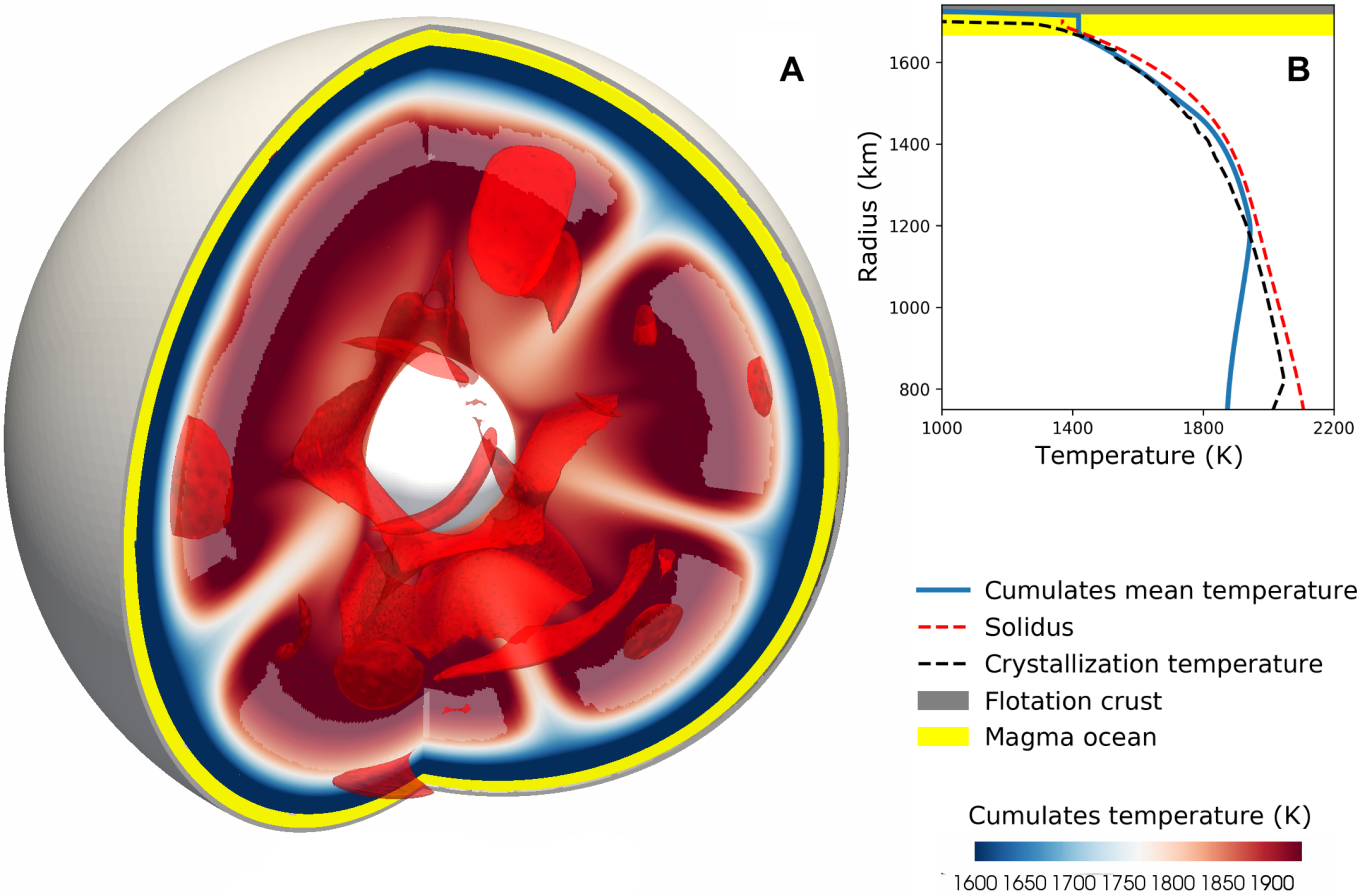
Thermal state of the Moon at 100 Ma. (**A**) Snapshot of the temperature field in the cumulates after 100 Ma for our fiducial case using *k*_crust_ = 2 W m^−1^ K^−1^ and η_ref_ = 10^21^ Pa s (Fig. 2, blue curve). Convection in the cumulates occurs while the magma ocean (yellow area) is still solidifying and the plagioclase crust (gray area) is still growing. (**B**) Corresponding laterally averaged temperature profile (blue line). In hot upwellings, the temperature exceeds the solidus [red dashed line in (B)], causing partial melting [pale areas in (A)] that results in heat piping.

A prolonged LMO solidification would likely allow solid-state convection in the cumulate layer (*11*–*13*) such that adiabatic decompression in rising upwellings could drive the temperature above the solidus, causing the cumulates to remelt. When these melts are extracted into the cooler overlying magma ocean, the heat flow from the cumulates to the LMO is enhanced, a mechanism called heat piping. Thus, we extended the previous model by simulating thermal convection in the growing cumulates in a three-dimensional (3D) spherical shell whose outer radius evolves as the magma ocean shrinks (Fig. 3) (*11*) and by parametrizing melt production in the convecting domain using the computed temperature field (see Materials and Methods). Thermal convection in the cumulates is controlled by their viscosity, which depends on composition, temperature, water content, and interstitial melts (*14*). To cover uncertainties in these parameters, we considered a standard temperature- and pressure-dependent diffusion creep rheology and varied the reference viscosity η_ref_ (at 1600 K and 3 GPa) over a broad range, from 10^19^ to 10^22^ Pa s, representing rocks ranging from very weak, and volatile-rich, to very stiff and dry.

For our fiducial value of *k*_crust_ = 2 W m^−1^ K^−1^, and for a reference viscosity η_ref_ = 10^22^ Pa s (Fig. 2B, orange line), the onset time of convection in the cumulates is longer than the LMO lifetime, meaning that cumulates perpetually remain in a conductive regime, yielding the same evolution as the purely conductive case (Fig. 2A, black dashed line). For lower values of η_ref_, solid-state convection starts before the LMO is fully solidified and results in heat piping, which causes transient thickening of the magma ocean. Although the reference viscosity correlates with the onset time of convection, the overall spread in the duration of LMO solidification stays within 40 Ma for all values of η_ref_. This reflects a trade-off between heat-piping flux and crustal thickness: for high η_ref_, the heat-piping flux is weak and starts late, below a thick and well-insulating crust (Fig. 2B, blue line); for low η_ref_ (Fig. 2B, cyan line), the heat-piping flux is stronger and begins earlier, when the crust is thin and heat is rapidly conducted away.

We also tested the influence of the overturn of a chemically dense and rheologically weak late-crystallizing layer (*15*–*17*) on the dynamics of the cumulates and on heat piping. The results show, however, that such an overturn does not have a significant effect on the overall solidification time scale of the LMO for our fiducial set of parameters. It can, therefore, be neglected in our fiducial case (see Materials and Methods).

Last, as the initial depth of the LMO is uncertain (*18*), we computed the crystallization sequence for three additional magma ocean depths, including a whole-mantle (1350-km-deep) and a 500-km-deep magma ocean [representing the two end members proposed in previous studies (*18*, *19*)], as well as for an intermediate case with a depth of 700 km. We used the corresponding crystallization temperatures and melting curves to simulate the magma ocean solidification for our fiducial set of parameters. The whole-mantle magma ocean, because it involves a larger part of the mantle, contains more heat-producing elements and thus solidifies over a longer time (242 Ma). Conversely, a 500-km-thick magma ocean has a lower internal heat production and a thinner final crust (~32 km) and, therefore, cools more rapidly. For our fiducial set of parameters, convection (i.e., heat piping) does not start in the cumulates before the end of solidification, after 56 Ma. For the same parameter values, convection starts early in the cumulates of a 700-km-deep magma ocean, resulting in sufficient heat piping to ultimately reach a similar LMO lifetime as obtained for the deeper magma oceans.

## DISCUSSION

### Comparison to chronology of lunar samples

The LMO solidification time scale of up to ~200 Ma inferred from our model seems inconsistent with the chronology of LMO products. Crystallization ages for ferroan anorthosites (FANs), representing the LMO’s flotation crust, range from ~100 to ~200 Ma after the beginning of the solar system. However, this range may not reflect true differences in formation ages, because some of these ages have large uncertainties and different chronometers have not yielded concordant results (*20*). A precise and concordant age has only been found for a single FAN sample (60025), which crystallized at 4.360 ± 0.003 Ga (*21*). This age is considered an important marker in lunar chronology, as it coincides with the two-stage model age for the isolation of the remaining small percentage of the LMO called urKREEP [for strong enrichments in potassium (K), rare-earth elements (REEs), and phosphorus (P)]. The urKREEP model age was determined using the ^147^Sm-^143^Nd and ^176^Lu-^176^Hf systematics of KREEP-rich samples having crystallization ages from ~4.35 to ~3.85 Ga. Back-projection of the initial Nd and Hf isotopic compositions of these samples to the chondritic composition (assumed to represent the bulk LMO) yields a model age for urKREEP formation of 4.368 ± 0.029 Ga (*22*). The close agreement of this age with the age of FAN 60025 has been used to argue for a rapid crystallization of the LMO at ~4.36 Ga (*20*, *22*). Because the LMO is thought to cool rapidly until the anorthositic crust formed, this age may also represent the Moon’s formation age (*20*, *22*).

**Fig. 4:**
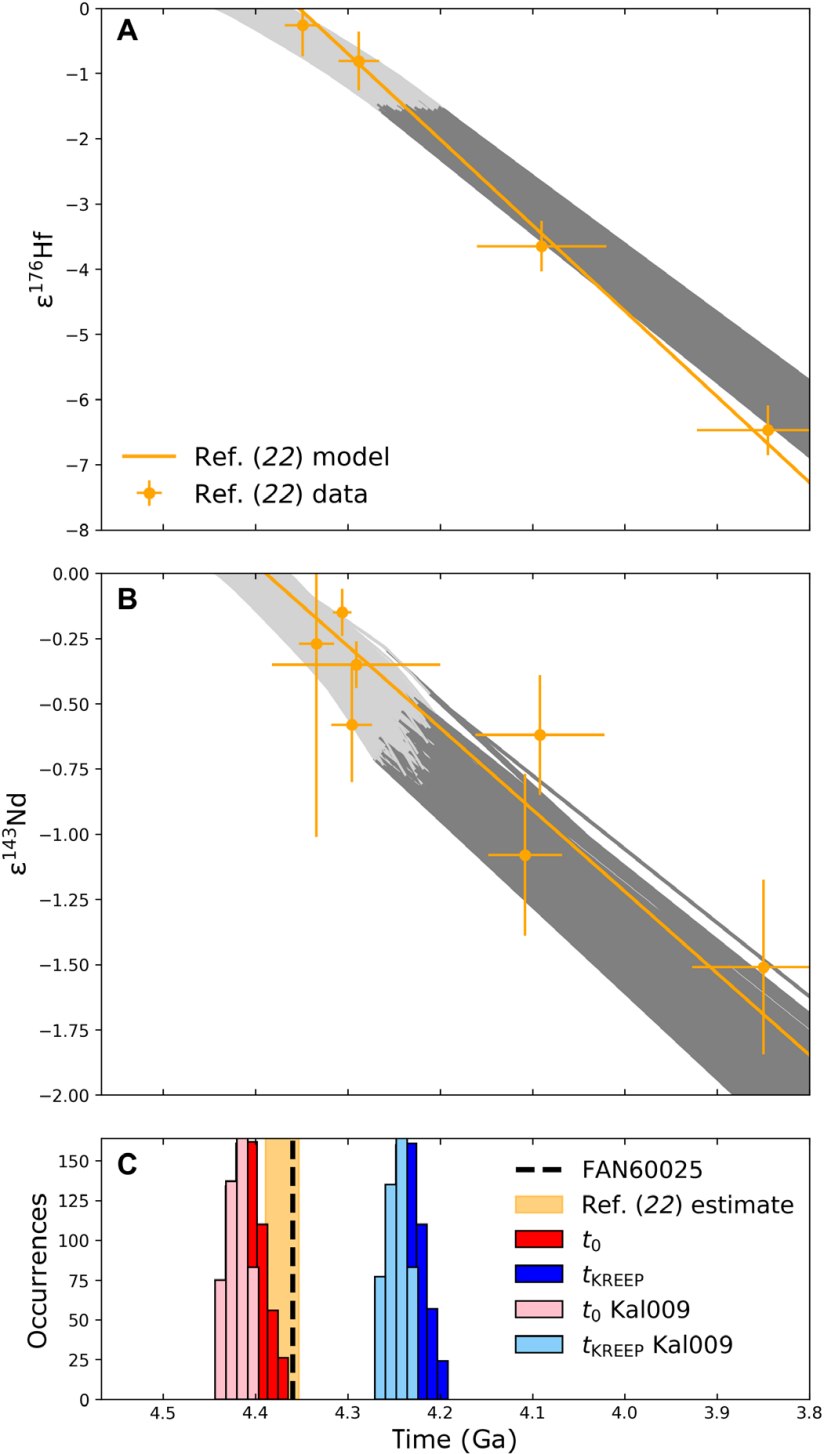
Isotopic systematics of the evolving LMO and KREEP. (**A**) Evolution of ε^176^Hf, ε^143^Nd (**B**) in the LMO and KREEP, and (**C**) distribution of Moon formation time (*t*_0_) and KREEP isolation time (*t*_KREEP_). Light gray curves represent the evolution in the magma ocean before KREEP formation; dark gray lines represent the subsequent KREEP decay lines obtained from Monte Carlo models that fitted at least 11 of the 12 data points for Lu-Hf and Sm-Nd systems used in (*22*) and plotted in orange (circles for KREEP basalts and diamonds for Mg-suite rocks). One KREEP basalt sample used for the Sm-Nd system (NWA773) has an age of 2.993 Ga and falls out of plot (B). The model ages of KREEP from (*22*) for the two systems [corresponding to the intercept of the orange solid lines, with the *x* axis corresponding to the extremes of the orange shaded area in (C)]. The age of FAN 60025 is represented by a black dashed line in (C). Among the cases shown on the histograms in (C), those compatible with Kal009 data are highlighted on the brighter subhistograms.

However, the model age for urKREEP formation calculated in this manner relies on the assumption that urKREEP derives from a reservoir with chondritic composition that underwent a single fractionation event. Yet, if LMO solidification took ~200 Ma, as in our fiducial case, there would have been significant isotopic evolution before the isolation of urKREEP. To assess this effect quantitatively, we calculated the isotopic evolution of the LMO based on our crystallization sequence and thermal evolution time series computed for our fiducial set of parameters. In the model, we treated the age of the Moon (*t*_0_), corresponding to the onset time of LMO solidification, as a free parameter. The urKREEP reservoir is assumed to form at time *t*_KREEP_, when the ^176^Lu/^177^Hf and ^147^Sm/^144^Nd ratios of the remaining LMO match those inferred for urKREEP (*21*). Figure 4 shows the results for the cases that successfully reproduce the Hf and Nd isotopic evolution of KREEP-rich samples (results are shown as ε^176^Hf and ε^143^Nd values, the parts-per-10^4^ deviation from chondritic compositions), demonstrating that at *t*_KREEP_ the LMO had subchondritic ε^176^Hf and ε^143^Nd. Moreover, the characteristic ^176^Lu/^177^Hf and ^147^Sm/^144^Nd of KREEP are reached between ~4.27 and ~4.19 Ga (Fig. 4C, blue histogram), i.e., 100 to 180 Ma later than previously inferred from the same data but assuming chondritic ε^176^Hf and ε^143^Nd at the time of urKREEP formation (*22*). Thus, contrary to previous conclusions, a long-lived LMO is consistent with the ages and isotopic composition of bulk KREEP-rich samples.

The Lu-Hf isotopic evolution of urKREEP has also been investigated using lunar zircons separated from KREEP-rich highland breccias (*23*). However, the zircon and bulk rock data cannot simultaneously be fitted in a single model, because they do not plot along a common isotopic evolution line for urKREEP. Also, the zircon data can only be fitted to the LMO’s Hf isotopic evolution for an unrealistically old onset time of LMO crystallization of 4.567 Ga (i.e., the age of the solar system). As such, and unlike for the bulk rock data, the zircon data are difficult to reconcile with a realistic model for the LMO’s thermal and isotopic evolution. One problem with the lunar zircon data may be that their Hf isotopic compositions require large downward corrections of ε^176^Hf for the effects of cosmic ray exposure (*23*). These corrections are inherently uncertain, and so, the lunar zircons may not accurately record the isotopic evolution of the LMO (see Materials and Methods).

### Age of the Moon

Our model not only provides the formation time of urKREEP but also predicts the time at which the LMO started to crystallize, which closely approximates the Moon’s formation time (that we treat as a free parameter). In the model above, the age of the Moon is 4.44 to 4.36 Ga (Fig. 4C, red histogram, and table S5). This age can be further refined by also including the well-dated lunar meteorite Kalahari 009, which has a precise age of 4.369 ± 0.007 Ga (*24*) and an elevated initial ε^176^Hf, indicating that it derived from a high-Lu/Hf source region (*25*). Our fractionation model shows that a pyroxene-rich reservoir that crystallized at 250 to 300 km depth has a sufficiently high Lu/Hf to reproduce the initial ε^176^Hf at the time of Kalahari 009’s crystallization (see Materials and Methods). Selecting the cases that successfully fit the data for KREEP-rich samples and Kalahari 009 results in more tightly constrained *t*_0_ and *t*_KREEP_, corresponding to a Moon formation age between 4.40 and 4.44 Ga and a KREEP formation age between 4.22 and 4.27 Ga (Fig. 4C).

**Fig. 5:**
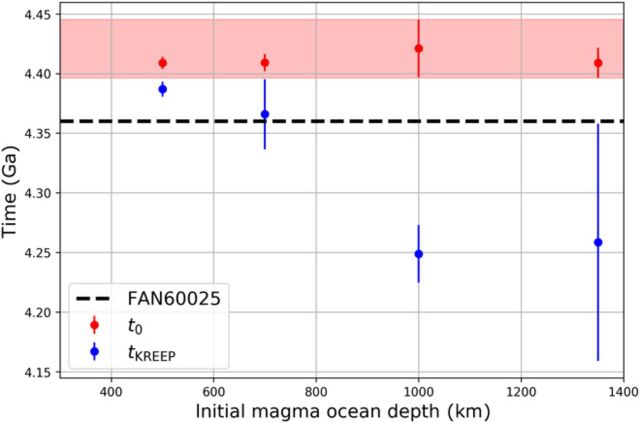
Distribution of Moon (*t*_0_, red points) and KREEP (*t*_KREEP_, blue points) formation times computed for the various initial LMO depths investigated. The red shaded area corresponds to our estimate range (4.40 to 4.45 Ga) for the Moon formation event. The black dashed line represents the age of FAN 60025 (*21*), consistent with the crust formation time span obtained for a 1000-km-deep LMO or a whole-mantle LMO.

While estimates of *t*_0_ and *t*_KREEP_ obtained for our fiducial set of parameters are arguably the most meaningful, it is nevertheless important to assess how these estimates change when a different set of parameters is used. As noted above, variations in the reference viscosity have limited impact due to the convergence of the time series (as seen in table S5). By contrast, the initial depth of the magma ocean has a stronger influence, leading to variations in the LMO’s lifetime that potentially translate into variations in *t*_0_ and *t*_KREEP_. Importantly though, *t*_0_ is less sensitive to variations in parameter space than *t*_KREEP_, and a longer LMO lifetime generally tends to delay *t*_KREEP_ rather than affect *t*_0_ (Fig. 5 and fig. S8). For instance, a 500-km-deep LMO, which crystallizes rapidly because it lacks heat piping, yields the oldest *t*_KREEP_, consistent with previous estimates based on the chondritic model of urKREEP (*22*). However, in this case, FAN 60025 would postdate the final solidification of the LMO, which is unrealistic unless this sample did not form as a flotation cumulate of the magma ocean. Although longer-lived, a 700-km-deep magma ocean yields only slightly younger urKREEP formation times, which overlap with the age of FAN 60025. Only for the 1000-km-deep and whole-mantle magma oceans is *t*_KREEP_ substantially younger than FAN 60025 and extends up to ~4.16 Ga. Altogether, investigating the complete parameter space of our model provides a robust estimate for the age of the Moon of between 4.40 and 4.45 Ga, and a KREEP formation time at least several tens of Ma later (~170 Ma for our fiducial case), and possibly as late as 4.16 Ga. One important implication of this timeline of lunar evolution is that FAN 60025, with its precise age of 4.360 ± 0.003 Ga, does not represent the earliest lunar crust, which started forming at 4.40 to 4.45 Ga; it rather samples later crust that formed during the prolonged crystallization of the LMO.

### Relation between age of the Moon and final differentiation of Earth

The 4.40 to 4.45 Ga age for the Moon obtained in our fiducial case agrees well with the U-Pb age of Earth of between ~4.45 and ~4.40 Ga (*26*–*29*). The U-Pb age, therefore, dates an event associated with the Moon-forming impact, such as segregation of Pb into Earth’s core (*29*), and not a later event such as the “late veneer” (the material added to Earth’s mantle after the Moon-forming impact) (*26*). Moreover, the inverse correlation between time of the last giant impact and mass of the late veneer (*30*) reveals that our inferred age of the Moon is consistent with estimates of the late veneer’s mass derived from the abundances of highly siderophile elements in Earth’s mantle (*30*). The age of the Moon determined here also coincides with the peak of 4.43 to 4.49 Ga ^40^Ar-^39^Ar degassing ages for meteorites from the asteroid belt, suggesting that this peak may reflect a surge in high-velocity impacts from ejecta resulting from the Moon-forming impact (*31*). The convergence of these independent estimates not only provides a robust and precise age for the Moon-forming impact but also consistently links this event to the differentiation of Earth and the dynamical evolution of the inner solar system.

## MATERIALS AND METHODS

### Crystallization model

The crystallization sequence of the magma ocean (fig. S1) is computed similarly to (*16*) using FXMOTR (*32*) in the deeper part of the mantle, where it is in good agreement with experimental data (*33*), and alphaMELTS (*34*) in the shallower part, where it provides better agreement with experimental data from the same set (*35*). For our fiducial case, we assume fractional crystallization of a spherical shell with an initial thickness of 1000 km (with an inner radius of 740 km and an outer radius of 1740 km), with 5% trapped melt in the cumulates, and a bulk lunar mantle composition from (*36*) for the major elements and (*37*) for the heat-producing trace elements (U, Th, and K). The oxygen fugacity is assumed to be constant at one log_10_ unit below the iron-wüstite buffer (IW-1). All minerals (except plagioclase) that form in a given temperature step are assumed to accumulate at the bottom of the LMO and equilibrate with the melt at the respective pressure conditions. The depth and bottom pressure of the LMO are updated after each crystallization step according to the volume of the crystallized cumulate. Plagioclase is assumed to float to the top of the magma ocean and form anorthositic crust. The thickness of the plagioclase crust at the end of crystallization is 44 km, in good agreement with estimates based on gravity and topography data (*38*).

The resulting crystallization temperature, solidus, heat-producing element content, and composition of the cumulate pile are shown in figs. S1 and S2. For the unmolten primitive lower mantle, we consider an accretion-like initial temperature profile, decreasing with depth from the crystallization temperature at the bottom of the magma ocean to the solidus of the bulk silicate Moon composition at the core-mantle boundary (CMB). This implies that the unmolten lower mantle is partly above its solidus, thus potentially causing partial melting. Nevertheless, we neglected the resulting heat piping effect that would occur at the very beginning of the magma ocean solidification because the heat would be efficiently extracted by the then very high surface heat flux.

The progressive enrichment in incompatible, cold crystallizing material in the LMO during solidification results in a strong temperature gradient at the very end of the solidification. The solidification of the last layer of ≈1 km of the LMO needs the cooling of several hundreds of K and is associated with the protracted tail in the crystallization time series on Fig. 2.

Similar to previous studies on LMO solidification (*2*, *3*), we consider the crystallization of an initially 1000-km-deep LMO. An initially whole-mantle LMO has also been proposed (*19*). This has two effects on our results: First, the crystallization temperature changes because fractionation occurs throughout the whole mantle rather than through the outermost 1000 km. Second, for the same reason, a whole-mantle LMO results in a higher enrichment of the LMO in heat-producing elements, because no heat-producing elements are sequestered in the unmolten lower mantle. Last, an initially thinner magma ocean has also been proposed (*18*). Therefore, we also computed the crystallization sequence and associated thermal evolution of initially 500- and 700-km-deep magma oceans. These LMOs have, conversely, a lower heat budget. Furthermore, because we also assume an accretion-like temperature in the unmolten lower mantle, their corresponding bulk silicate Moon average temperature is lower, which translates into less efficient convection due to the temperature dependence of the rheology. This results in an absence of heat-piping effect for the shallowest LMO using our fiducial set of parameters.

### Thermal evolution model

#### *The core*

The core is treated as an isothermal sphere of temperature *T*_core_. The equation controlling the time evolution of *T*_core_ reads

$$c_{p,\text{core}}\rho_{\text{core}}V_{\text{core}}\frac{{\mathrm{dT}}_{\text{core}}}{\mathrm{dt}}=-S_{\text{CMB}}q_{\text{CM}B}$$ (1)

where *c*_p, core_ is the core heat capacity, ρ_core_ is the core density, $V_{\text{core}}=4/3\pi R_{\text{core}}^{3}$ is the core volume (*R*_core_ being the core radius), *t* is the time, $S_{\text{CMB}}=4\pi R_{\text{core}}^{2}$ is the surface of the CMB, and *q*_CMB_ is the heat flux at the CMB, which is computed as

$$q_{\text{CMB}}=k{\frac{\partial T}{\partial r}|}_{r=R_{\text{core}}}$$ (2)

where *k* is the thermal conductivity of the solid mantle and ${\frac{\partial T}{\partial r}|}_{r=R_{\text{core}}}$ is the average temperature gradient above the core.

#### *The solid cumulates*

The temperature of the solid cumulates is modeled in two different ways. When only the effect of the thermal conductivity of the crust is considered, it follows a 1D time-dependent heat diffusion equation. When the effect of heat piping is considered, it is modeled by 3D thermal convection.

##### 1D heat diffusion

We model the thermal evolution of the solid cumulates by solving the heat diffusion equation in a 1D spherically symmetric shell of constant inner radius *R*_core_ and outer radius *R*_MO_, corresponding to the radius of the bottom of the magma ocean

$$\frac{\partial T}{\partial t}=\frac{2}{r}\kappa \frac{\partial T}{\partial r}+\kappa \frac{\partial^{2}T}{\partial r^{2}}+\frac{h}{c_{p}}$$ (3)

where κ = *k*/(ρ*c*_p_) is the thermal diffusivity, ρ is the mantle density, *c*_p_ is the heat capacity, and *h* is the internal heat production per unit mass. The initial internal heating *h* is the volume average of the heat production in the solid cumulates at the initial time of the simulations (corresponding to a 110-km-deep magma ocean). It is computed from our crystallization sequence accounting for the partitioning of the long-lived radionuclides ^235^U, ^238^U, ^232^Th, and ^40^K. The internal heating’s decay is computed using the decay constants and isotopic ratios from (*37*) (computed at 4.567 Ga). The boundary conditions for Eq. 3 are as follows: *T*|_*r*=*R*_core__ = *T*_core_ and *T*|_*r*=*R*_MO__ = *T*_MO_, where *T*_MO_ is the magma ocean’s temperature. Equation 3 is solved using an implicit time stepping and a first-order central finite-difference scheme on a 100-point regular grid. Because the geometry evolves as the crust grows, the position of the grid points changes from one time step to the next. To account for this, the temperature profile (before solving Eq. 3) is interpolated from the profile at the previous time step onto the new grid.

##### 3D thermal convection

To compute secondary melting caused by solid-state convection in the solid cumulates, we use the finite-volume code GAIA (*38*) to solve the equations of thermal convection in a 3D spherical shell composed of 132 shells containing 10,242 computation nodes each (see section S1.2 for a discussion about the choice of the resolution). The inner radius of the domain corresponds to *R*_core_, while the outer radius evolves following *R*_MO_ as described in (*11*).

We solve the conservation of mass, linear momentum, and thermal energy with the Boussinesq approximation as follows

$$\vec{\nabla }\cdot \vec{v}=0$$ (4)

$$\vec{\nabla }\cdot (\eta (\vec{\nabla }\vec{v}+\vec{\nabla }{\vec{v}}^{T}))-\vec{\nabla }p=\rho g(r)\alpha (T-T_{s})\vec{e_{r}}$$ (5)

$$\frac{\partial T}{\partial t}+\vec{v}\cdot \vec{\nabla }T-\kappa \nabla^{2}T=\frac{h}{c_{p}}$$ (6)

where $\vec{v}$ is the velocity, η is the dynamic viscosity, *p* is the dynamic pressure, *g*(*r*) is the radially dependent gravity acceleration, α is the thermal expansivity, *T*_s_= 250 K is the surface temperature, and $\vec{e_{r}}$ is the unitary radial vector. The viscosity is temperature and pressure dependent, following an Arrhenius law

$$\eta =\eta_{\text{ref}}(\frac{E^{*}+p_{\mathrm{ls}}V^{*}}{\mathrm{RT}}-\frac{E^{*}+p_{\text{ref}}V^{*}}{{\mathrm{RT}}_{\text{ref}}})$$ (7)

where η_ref_ is the reference viscosity, i.e., the viscosity at the reference pressure *p*_ref_ = 3 GPa and temperature *T*_ref_ = 1600 K; E* = 335 kJ/mol and V* = 4 × 10^−6^ m^3^/mol are the activation energy and volume, respectively, characteristic of olivine diffusion creep (*14*); *p*_ls_ is the lithostatic pressure; and *R* is the gas constant.

Because of its small core, the gravity acceleration in the lunar mantle is not uniform throughout the mantle. We compute *g* by considering a constant density ρ, resulting in

$$g(r)=\frac{4/3\pi G}{r^{3}}(\rho_{\text{core}}R_{\text{core}}^{3}-\rho (r^{3}-R_{\text{core}}^{3}))$$ (8)

where *G* is the gravitational constant. The internal heating per unit mass and the thermal boundary conditions associated to this system are the same as those introduced in the previous section. The dynamical boundary conditions are free slip at both inner and outer boundaries.

##### 2D thermo-chemical convection

For some cases, we followed (*16*) to assess the buoyancy effects of a late-crystallizing, dense, and weak layer at the top of the cumulates. This layer, which simulates ilmenite-bearing cumulates of density ρ_1_ = 3775 kg/m^3^ as computed from our crystallization model, is taken into account by solving the advection equation

$$\frac{\partial C}{\partial t}+\vec{v}\cdot \vec{\nabla }C=0$$ (9)

where *C* is the nondimensional chemical density field, whose value is one in the dense cumulates (a layer at the top of the cumulate pile of thickness *D*_1_= 27 km) and zero elsewhere. Equation 9 is implemented using the particle-in-cell method as described in (*39*) and applied in the context of thermochemical convection in the early Moon by (*16*). Because of the prohibitively high computational cost of using the particle-in-cell method with a high-resolution 3D grid, we ran these simulations on a 2D cylindrical grid, which allowed us to adopt a radial resolution of 249 shells. Compositional buoyancy is accounted for by adding a body-force term in Eq. 5, which now reads

$$\vec{\nabla \cdot }(\eta (\vec{\nabla }\vec{v}+\vec{\nabla }{\vec{v}}^{T}))-\vec{\nabla }p=(\rho g(r)\alpha (T-T_{s})-\rho_{1}g(r)C)\vec{e_{r}}$$ (10)

The weaker rheology of the dense layer is accounted for by modifying the viscosity field as follows

$$\eta =\eta_{A}\Delta \eta^{C}$$ (11)

where η_A_ is the Arrhenius viscosity computed from Eq. 7, Δη is the viscosity ratio between the weak layer and the average mantle, and *C* is the concentration of weak material.

Last, the dense layer is also enriched in heat-producing elements. Its initial rate of internal heating (computed from our crystallization model) is *h*_1_ = 1.27 × 10^−10^ W/m^2^. The composition-dependent internal heating rate hence reads

$$h=(1-C)h_{0}+{\mathrm{Ch}}_{1}$$ (12)

##### Heat piping

We assume that extraction of the secondary melts due to decompression melting in the rising upwellings of convecting solid cumulates buffers the maximum temperature in the cumulates to the solidus. After solving Eq. 6, if the temperature locally exceeds the solidus, a mass of melt *dM*_melt_ is produced locally, driving the temperature back to the solidus through consumption of latent heat of melting

$${\mathrm{dM}}_{\text{melt}}=\frac{1}{L}\rho c_{p}\mathrm{dV}\;\text{max}(T-T_{\text{sol}},0)$$ (13)

where *L* is the latent heat of melting, *dV* is the volume on which the mass of melt is computed, and *T*_sol_ is the local solidus temperature. This mass of melt is then extracted at its solidus temperature, into the magma ocean, providing a heat source proportional to the temperature difference between the magma ocean and the solidus at the melting position, resulting in a total heat source *dE*_HP_

$${\mathrm{dE}}_{\mathrm{HP}}=\int_{V_{\text{cumulates}}}c_{p}(T_{\text{so}l}-T_{\text{MO}})dM_{\text{melt}}$$ (14)

#### *Magma ocean*

The magma ocean is modeled as an isothermal spherical shell of inner radius *R*_MO_ and outer radius *R*_crust_ (the radius of the bottom of the flotation crust). We solve the conservation of heat in the magma ocean by balancing the different fluxes in and out of the magma ocean with its internal heating. The heat conservation equation for the thermal energy of the magma ocean reads

$$\begin{array}{c} \rho c_{p}\frac{d}{\mathrm{dt}}(\int_{V_{\text{MO}}}T_{\text{MO}}\mathrm{dV})=q_{\text{bot}}S_{\text{bot}}-q_{\text{top}}S_{\text{top}}+\\ \rho h_{\text{MO}}V_{\text{MO}}+\frac{{\mathrm{dE}}_{\text{HP}}}{\mathrm{dt}}+\rho L\frac{{\mathrm{dV}}_{\text{MO}}}{\mathrm{dt}}+\rho c_{p}\frac{{\mathrm{dV}}_{\text{MO}}}{\mathrm{dt}}T_{\text{MO}} \end{array}$$ (15)

where *q*_bot_ and *q*_top_ are the conductive heat fluxes at the bottom and the top of the magma ocean, respectively; *q*_bot_ and *q*_top_ are the surfaces of the bottom and the top of the magma ocean, respectively; and *T*_MO_, *h*_MO_, and *V*_MO_ are the magma ocean’s temperature, internal heating per unit mass, and volume, respectively. The left-hand side term corresponds to the variation in thermal energy of the magma ocean (due to variation in both temperature and volume because it is an open system). The first two terms on the right-hand side correspond to the conductive heat fluxes at the top of the cumulates into the magma ocean and at the base of the crust out of the magma ocean and are computed as $q_{\text{bot}}=-k{\frac{\partial T}{\partial r}|}_{r=R_{\text{MO}}}$ and $q_{\text{top}}=-k{\frac{\partial T}{\partial r}|}_{r=R_{\text{crust}}}$, where $S_{\text{bot}}=4\pi R_{\text{MO}}^{2}$ and $S_{\text{top}}=4\pi R_{\text{crust}}^{2}$ are the surfaces of the bottom and top of the magma ocean, and *k*_crust_ is the thermal conductivity of the crust. The third term on the right side of Eq. 15 corresponds to the internal heating of the magma ocean. Note that *h*_MO_ depends on time, due to the decay of heat-producing elements, and on *R*_MO_, due to the enrichment in incompatible heat-producing elements as the magma ocean crystallizes. The fourth term is the heat-piping flux introduced in the previous section. The fifth term corresponds to the release of latent heat upon crystallization (or consumption of latent heat upon melting). Last, the sixth term corresponds to the heat flow due to the mass flux out of the magma ocean of the settling crystals. This term must appear because we consider the heat conservation of an open system.

Because the magma ocean is assumed to be isothermal, we can expand the left-hand side term as

$$\frac{d}{\mathrm{dt}}(\int_{V_{\text{MO}}}T_{\text{MO}}\mathrm{dV})=T_{\text{MO}}\frac{dV_{\text{MO}}}{\mathrm{dt}}+V_{\text{MO}}\frac{dT_{\text{MO}}}{\mathrm{dt}}$$ (16)

and cancels out the first term on the right-hand side of Eq. 16 with the last term on the right-hand side of Eq. 15. We can also write the latent heat term as

$$\rho L\frac{{\mathrm{dV}}_{\text{MO}}}{\mathrm{dt}}=\rho L\frac{{\mathrm{dV}}_{\text{MO}}}{{\mathrm{dR}}_{\text{MO}}}\frac{{\mathrm{dR}}_{\text{MO}}}{{\mathrm{dT}}_{\text{MO}}}\frac{{\mathrm{dT}}_{\text{MO}}}{\mathrm{dt}}$$ (17)

Note that $\frac{dV_{\text{MO}}}{dR_{\text{MO}}}$ is not simply $-4\pi R_{\text{MO}}^{2}$ due to the coupled evolution of *R*_MO_ and *R*_crust_ defining its boundaries (see Eq. 23). Furthermore, both the evolution of *T*_MO_ and *R*_MO_ are linked because *T*_MO_ follows the radial profile of *T*_crys_ as the magma ocean crystallizes

$$\frac{dT_{\text{MO}}}{{\mathrm{dR}}_{\text{MO}}}={\frac{dT_{\text{crys}}}{\mathrm{dr}}|}_{r=R_{\text{MO}}}$$ (18)

Therefore, inserting Eq. 18 into Eq. 17, we can write the latent heat term of Eq. 15 as

$$\rho L\frac{{\mathrm{dV}}_{\text{MO}}}{\mathrm{dt}}=\rho L\frac{{\mathrm{dV}}_{\text{MO}}}{{\mathrm{dR}}_{\text{MO}}}{\frac{{\mathrm{dT}}_{\text{crys}}}{\mathrm{dr}}|}_{r=R_{\text{MO}}}^{-1}\frac{{\mathrm{dT}}_{\text{MO}}}{\mathrm{dt}}$$ (19)

Last, inserting Eqs. 16 and 17 into Eq. 15, we obtain the equation that controls the evolution of the magma ocean temperature

$$\rho c_{p}V_{\text{MO}}\frac{{\mathrm{dT}}_{\text{MO}}}{\mathrm{dt}}=\frac{S_{\text{bot}}q_{\text{bo}t}-S_{\text{top}}q_{\text{top}}+\rho h_{\text{MO}}V_{\text{MO}}+\frac{{\mathrm{dE}}_{\text{HP}}}{\mathrm{dt}}}{1-\frac{L}{c_{p}}\frac{dV_{\text{MO}}}{{\mathrm{dR}}_{\text{MO}}}{\frac{{\mathrm{dT}}_{\text{crys}}}{\mathrm{dr}}|}_{r=R_{\text{MO}}}^{-1}}$$ (20)

#### *Flotation crust*

The flotation crust is modeled as a spherically symmetric shell of inner radius *R*_crust_ and outer radius *R*_Moon_, whose temperature distribution is obtained solving the 1D heat diffusion equation, including heat sources

$$\frac{\partial T}{\partial t}=\frac{2}{r}\kappa_{\text{crust}}\frac{\partial T}{\partial r}+\kappa_{\text{crus}t}\frac{\partial^{2}T}{\partial r^{2}}+\frac{h_{\text{crust}}}{c_{p}}$$ (21)

where κ_crust_ = *k*_crust_/(ρ_crust_*c*_p_) is the thermal diffusivity of the crust and *h*_crust_ is its heat production per unit mass. The time dependence of the geometry is handled the same way as described for Eq. 3, with the difference that, in the case of the crust, the outer radius is fixed, while the inner radius evolves with time.

The crust starts from a thickness of 5 km as in (*2*). The time evolution of *R*_crust_ is obtained by assuming a constant ratio *f* of volume of crystallized plagioclase per unit volume of crystallized magma ocean and assuming that all plagioclase crystals formed settle at the top of the magma ocean. The value of *f* is imposed by the thickness of the crust at the end of crystallization (44 km as obtained from our model). By definition, we have

$$f=-\frac{{\mathrm{dV}}_{\text{crust}}}{{\mathrm{dV}}_{\text{MO}}}=\frac{\frac{{\mathrm{dV}}_{\text{crust}}}{{\mathrm{dR}}_{\text{crust}}}{\mathrm{dR}}_{\text{crust}}}{{\left(\frac{{\mathrm{dV}}_{\text{MO}}}{{\mathrm{dR}}_{\text{MO}}}\right)}_{R_{\text{crust}}}{\mathrm{dR}}_{\text{MO}}+{\left(\frac{{\mathrm{dV}}_{\text{MO}}}{{\mathrm{dR}}_{\text{crust}}}\right)}_{R_{\text{MO}}}{\mathrm{dR}}_{\text{crust}}}$$ (22)

where ${\left(\frac{dV_{\text{MO}}}{dR_{\text{MO}}}\right)}_{R_{\text{crust}}}$ is the derivative of *V*_MO_ in *R*_MO_ when *R*_crust_ is kept constant and inversely for ${\left(\frac{dV_{\text{MO}}}{dR_{\text{crust}}}\right)}_{R_{\text{MO}}}$. Noting that ${\left(\frac{dV_{\text{MO}}}{dR_{\text{crust}}}\right)}_{R_{\text{MO}}}=-\frac{dV_{\text{crust}}}{dR_{\text{crust}}}$, we can write Eq. 22 as

$${\mathrm{dR}}_{\text{crust}}=\frac{f}{1-f}\frac{{\left(\frac{{\mathrm{dV}}_{\text{MO}}}{{\mathrm{dR}}_{\text{MO}}}\right)}_{R_{\text{crust}}}}{{\left(\frac{{\mathrm{dV}}_{\text{MO}}}{{\mathrm{dR}}_{\text{crust}}}\right)}_{R_{\text{MO}}}}{\mathrm{dR}}_{\text{MO}}$$ (23)

which is the evolution for *R*_crust_. Equation 23 also yields

$$\frac{{\mathrm{dV}}_{\text{MO}}}{{\mathrm{dR}}_{\text{MO}}}={\left(\frac{{\mathrm{dV}}_{\text{MO}}}{{\mathrm{dR}}_{\text{MO}}}\right)}_{R_{\text{crus}t}}(1+\frac{f}{1-f})$$ (24)

#### *Initial setup*

The thermal evolution simulations start when the crust is 5 km thick (*R*_crust,0_ = 1735 km). For our fiducial initial depth of the LMO (1000 km), the remaining magma ocean is then 110 km deep (*R*_MO,0_= 1630 km) and its temperature is *T*_MO,0_= 1518 K. The initial temperature profile in the crust is linear from the bottom of the crust at temperature *T*_MO,0_ up to the surface at 250 K. As the magma ocean’s temperature evolves, its bottom radius evolves accordingly following the crystallization temperature profile.

### Isotope fractionation model

#### *Magma ocean isotopic signature*

The equation for chemical equilibrium of species *X* (*X* being either Lu, Hf, Sm, or Nd; see table S2 for values relative to these isotopic systems) between the magma ocean and the different crystallizing minerals reads ${\left[X\right]}_{\text{MO}}=1/K_{i}^{X}{\left[X\right]}_{i}$, where [*X*]_MO_ is the concentration of *X* in the magma ocean, [*X*]*_i_* is the concentration of *X* in mineral *i*, and $K_{i}^{X}$ is the partition coefficient of *X* between melt and mineral *i* (see table S3 for the list of minerals in the crystallization sequence). The minerals in the cumulate pile as well as plagioclase are assumed to be in equilibrium with the magma ocean only at the time step during which they crystallize and then to retain their crystallization concentration in *X* (unless modified by radioactive decay). The mass conservation of *X* between times *t* and *t* + *dt* [corresponding to a volume change *dV*_MO_ = (*V*_MO_(*t* + *dt*) − *V*_MO_(*t*)) of the magma ocean volume and a volume increase of the cumulates and the crust combined: *dV* = − *dV*_MO_] reads

$$\begin{array}{c} V_{\text{MO}}(t+\mathrm{dt}){\left[X\right]}_{\text{MO}}(t+\mathrm{dt})+[(1-\phi_{t})\sum_{i}C_{i}{\left[X\right]}_{i}(t+\mathrm{dt})\mathrm{dV}+\\ \phi_{t}{\left[X\right]}_{\text{MO}}(t+\mathrm{dt})\mathrm{dV}]=V_{\text{MO}}(t){\left[X\right]}_{\text{MO}}(t) \end{array}$$ (25)

where ϕ_t_ is the amount of trapped melt remaining in the cumulate pile and *C*_i_ is the abundance of mineral *i* in the layer of volume *dV* of cumulates formed during *dt*. The first term on the left-hand side represents the total amount of *X* in the magma ocean at time *t* + *dt*, the second term on the left-hand side is the amount of *X* that enters the different minerals of the newly crystallized cumulates and crust layer, and the right-hand side represents the total amount of *X* in the magma ocean at time *t*. Using the partition coefficient $K_{i}^{X}$, [*X*]_i_ can be replaced in Eq. 25 by $K_{i}^{X}{\left[X\right]}_{\text{MO}}$. If we write successively Eq. 25 for *X* = *P* (the radiogenic parent species, i.e., ^176^Lu or ^147^Sm) and for *X* = *D*_s_ (the stable daughter species, i.e., ^176^Hf or ^143^Nd), and divide the former by the latter, we obtain the isotopic ratio updated for fractionation before radioactive decay

$$\begin{array}{c} {\frac{\left[\tilde{P}\right]}{\left[D_{s}\right]}|}_{\text{MO}}(t+\mathrm{dt})={\frac{\left[P\right]}{\left[D_{s}\right]}|}_{\text{MO}}(t)\\ \frac{V_{\text{MO}}(t+\mathrm{dt})+((1-\phi_{t})\sum_{i}C_{i}K_{i}^{D}+\phi_{t})\mathrm{dV}}{V_{\text{MO}}(t+\mathrm{dt})+((1-\phi_{t})\sum_{i}C_{i}K_{i}^{P}+\phi_{t})\mathrm{dV}} \end{array}$$ (26)

Because the radiogenic and stable daughter species *D*_r_ and *D*_s_ have the same partition coefficient, the ratio [*D*_r_]/[*D*_s_] is not affected by fractionation. Equation 26 provides the first half of the time step. We then apply radioactive decay

$${\frac{\left[P\right]}{\left[D_{s}\right]}|}_{\text{MO}}(t+\mathrm{dt})={\frac{\left[\tilde{P}\right]}{\left[D_{s}\right]}|}_{\text{MO}}(t+\mathrm{dt})\text{exp}(-\mathrm{dt}\;\text{ln}(2)/\tau_{1/2})$$ (27)

$$\begin{array}{c} {\frac{\left[D_{r}\right]}{\left[D_{s}\right]}|}_{\text{MO}}(t+\mathrm{dt})={\frac{\left[D_{r}\right]}{\left[D_{s}\right]}|}_{\text{MO}}(t)+{\frac{\left[\tilde{P}\right]}{\left[D_{s}\right]}|}_{\text{MO}}(t+\mathrm{dt})\\ \left(1-\text{exp}(-\mathrm{dt}\;\text{ln}(2)/\tau_{1/2})\right) \end{array}$$ (28)

where τ_1/2_ is the half-life of the parent species. The magma ocean fractionation starts at a given time *t*_0_. The initial magma ocean has a chondritic composition for trace elements [from (*40*)]. The crystallization before crust formation is assumed to be instantaneous, and only fractionation is computed during this phase. We then iterate the complete procedure (including decay) using the time series for *V*_MO_ and *R*_MO_ (i.e., the crystallization front’s depth) from our simulations until ([*P*]/[*D*_s_])_MO_ reaches the value measured in KREEP samples from (*22*) for both systems at the same time ([^176^Lu]/[^177^Hf] = 0.0153 ± 0.0033 and [^147^Sm]/[^143^Nd] = 0.1723 ± 0.0019). If these ratios are never reached simultaneously, we discard the simulation. If they do, we consider the isotopic ratios of the remaining magma ocean as representative of the bulk KREEP ratios and compute their evolution due to decay.

#### *Cumulate whole-rock isotopic signature*

We also compute the whole-rock isotopic ratios in the cumulate pile forming before the growth of the flotation crust. We can thus make two simplification: First, all minerals forming settle in the cumulate pile (i.e., *C*_Plagioclase_ = 0); second, radioactive decay of long-lived species can be neglected [i.e., ${\left([D_{r}]/[D_{s}]\right)}_{\text{MO}}={\left([D_{r}\tilde{]/[}D_{s}]\right)}_{\text{MO}}$ and ${\left([P]/[D_{s}]\right)}_{\text{MO}}={\left([P\tilde{]/[}D_{s}]\right)}_{\text{MO}}$]. The whole-rock isotopic ratios in the cumulate pile at the time of crystallization (*t*_crys_) hence read

$${\frac{\left[P\right]}{\left[D_{s}\right]}|}_{WR}(t_{\text{crys}})=\frac{\sum_{i}C_{i}K_{i}^{P}}{\sum_{i}C_{i}K_{i}^{D}}{\frac{\left[P\right]}{\left[D_{s}\right]}|}_{\text{MO}}(t_{\text{crys}})$$ (29)

$${\frac{\left[D_{r}\right]}{\left[D_{s}\right]}|}_{WR}(t_{\text{crys}})={\frac{\left[D_{r}\right]}{\left[D_{s}\right]}|}_{\text{MO}}(t_{\text{crys}})$$ (30)

The ratios at any time *t* can then be computed using a simple radioactive decay law

$${\frac{\left[P\right]}{\left[D_{s}\right]}|}_{WR}(t)={\frac{\left[P\right]}{\left[D_{s}\right]}|}_{WR}(t_{\text{crys}})(1-\text{exp}((t-t_{\text{crys}})\text{ln}\;(2)/\tau_{1/2}))$$ (31)

$${\frac{\left[D_{r}\right]}{\left[D_{s}\right]}|}_{WR}(t)={\frac{\left[D_{r}\right]}{\left[D_{s}\right]}|}_{WR}(t_{\text{crys}})+({\frac{\left[P\right]}{\left[D_{s}\right]}|}_{WR}(t_{\text{crys}})-{\frac{\left[P\right]}{\left[D_{s}\right]}|}_{WR}(t))$$ (32)

Using this method, we compute the ε^176^Hf in the early formed cumulates, which are then discussed in relation with Kal009 (see section S4).

Once the crust starts growing, radioactive delay is not anymore, and Eq. 29 needs to be followed by Eqs. 27 and 28 applied to the whole-rock isotopic ratios. Because plagioclase does not enter the cumulate pile but the flotation crust, we still consider *C*_Plagioclase_ = 0.

#### *Monte-Carlo simulations*

Performing random sampling of both *t*_0_ (within the first 400 Ma of the solar system) and of the melt/rock partition coefficients of the cumulates minerals within the error bars from the literature (see table S3), we run 10^4^ fractionation models and select those for which the KREEP decay lines cross most data points (within the uncertainty ellipsoid). The data points are those used in (*22*) and are provided in table S4. The eight samples listed provide a total of 12 data points (four having both ε^143^Nd and ε^176^Hf values and four having only ε^143^Nd values). Some data points are not compatible with the others in that they cannot be all matched with a single line whose slope is given by the [^176^Lu]/[^177^Hf] and [^147^Sm]/[^143^Nd] ratios. Hence, the best fit consists in matching 11 of 12 data points. Those cases that reach the optimal fit are shown in Fig. 4.

## Supplementary Material

aba8949_SM.pdf

## SUPPLEMENTARY MATERIALS

Supplementary material for this article is available at http://advances.sciencemag.org/cgi/content/full/6/28/eaba8949/DC1
