# Supplementary material for: A long-lived magma ocean on a young Moon
Source: Sci Adv. 2020 Jul 10;6(28):eaba8949. doi: 10.1126/sciadv.aba8949 (PMC7351470; doi:10.1126/sciadv.aba8949)
Supplement: aba8949_SM.pdf [file aba8949_SM.pdf]

Science Advances
AAAS

advances.sciencemag.org/cgi/content/full/6/28/eaba8949/DC1

# Supplementary Materials for

## A long-lived magma ocean on a young Moon

M. Maurice*, N. Tosi, S. Schwinger, D. Breuer, T. Kleine

*Corresponding author. Email: maximemaurice@protonmail.com

Published 10 July 2020, *Sci. Adv.* **6**, eaba8949 (2020)
DOI: 10.1126/sciadv.aba8949

**This PDF file includes:**

Supplementary text
Figs. S1 to S9
Tables S1 to S5
References

## Supplementary Text

### 1. Thermal model benchmark

#### 1.1. Comparison with existing models

To benchmark our model, we reproduced the simulations performed in (*2*) and (*3*). One of the most important features of the crystallization model is the crystallization temperature, noted $T'_{crys}$ to distinguish it from the crystallization temperature from our model, and which reads in ref. (*2*):

$$\boldsymbol{T'_{crys}(r) = (-1.3714 \times 10^{-4})r^2 - 0.1724r + 2134 - 4.4\left(0.2V_{liq}(r) + 0.01\right)^{-1}}, \quad (1)$$

where $T'_{crys}$ is in K, $r$ is the radius in km and $V_{liq}(r) = V_{MO}(R_{MO} = r)/V_{MO,0}$ with $V_{MO,0}$ the initial volume of the magma ocean. Using a surface temperature of 25 K, as done in (*2*) and (*3*), we reach a final volume of the magma ocean of 1% (which is their criteria for the end of solidification) in 28 Myr. This is more than the ≈10 Myr found in (*2*) but very similar to the 26 Myr obtained for the same set-up in (*3*), where this discrepancy was already pointed out.

1.2. Convergence tests for the convection simulations

3-D convection simulations are computationally extremely costly, and can only be performed using relatively coarse grids. In order to choose a resolution that both allows for a reasonable computation time and yields a solution that faithfully captures the dynamics of the system (especially in terms of the top heat flux and the heat piping flux, which are the main outputs of the convection model), we ran convergence tests on 2-D cylindrical grids having 132, 191, 220 and 249 regularly spaced radial points (with respectively 654, 947, 1091 and 1234 points per shell), using our fiducial value for $k_{crust}$ (2 W/m/K) and the lowest value for $\eta_{ref}$ ($10^{19}$ Pa s). These resolutions were chosen to optimize the interpolation of the final gradient of the crystallization temperature profile, important during the final stage of the solidification (see Supplementary text section 1.3.1). Time-series of the depths of the magma ocean and crust, and of the cumulates and heat piping fluxes are plotted in Figure S3. They clearly show that lowering the resolution to 132 radial shells does not result in significant changes in the time-series (the final times agree within 5 Myr, i.e. 2%). We therefore chose to run our 3-D simulations with a radial resolution of 132 shells.

We also evaluated the discrepancy between 2-D and 3-D simulations since some tests (such as the thermo-chemical ones presented in Supplementary text section 2) were run on 2-D grids only. Discrepancies in the heat flow and temperature between 2-D cylindrical and 3-D spherical simulations are well documented in steady-state e.g. (*41*), but less constrained for transient regimes such as those considered here. A common method to mimic a flow in 3-D spherical shells using a 2-D cylindrical shell is to re-scale the core radius in order to recover in 2-D the 3-D ratio of core-mantle boundary surface to planet surface (*42*). This results in a decrease of the radius of the core,

which is already quite small for the Moon. Using our fiducial set-up, we compared three cases: a 3-D simulation using a resolution of 132 radial shells (i.e. the geometry used for the main results of the study), a 2-D simulation with "real" core radius and 249 radial shells, which closely matches the simulation computed on a 2-D grid with 132 shells (Figure S3), and another 2-D simulation using 249 shells, but with a re-scaled core. Figure S4 presents the time-series corresponding to these three cases. The 3-D simulation (green lines) yields the same onset time of heat piping as the 2-D simulation with non-scaled core (blue lines), but a slightly shorter magma ocean duration due to a lower heat-piping flux. The 2-D simulation with a re-scaled core (red lines) exhibits a later onset of convection, but a similar magma ocean lifetime as the non-scaled 2-D simulation due to the trade-off between late onset of heat-piping below thick crust and early onset below a thin crust, i.e. the mechanism described in the main text. This suggests that there is no need to rescale the core size in our 2-D runs, but to keep in mind that they likely slightly overestimate the duration of the magma ocean solidification.

### 1.3. Comparison between different effects included in the thermal model

The right-hand-side of equation [20] contains various terms. Some of them are necessary for the physical consistency of the model (like the heat flux through the crust which is necessary to ensure that the magma ocean cools), others have been considered in previous studies (like the latent heat term or the internal heating of the magma ocean), while others have not been considered before (like the conductive heat flux from the cumulates and the heat piping effect). While the present work focuses on the top heat flux and heat piping, this section presents a comparison of the influence of the other terms.

### 1.3.1. Typical heat fluxes and sources evolution

Figure S5 presents, for our fiducial case ($k_{crust}$ = 2 W/m/K and $\eta_{ref}$ = $10^{21}$ Pa s), the evolution of the various heat fluxes and sources at play along with the evolution of the temperature of the LMO and the depth of its top and bottom boundaries. At the beginning of the simulations, the conductive heat flow through the crust dominates because the crust is still very thin (5 km), but it decreases rapidly as the crust thickens and the magma ocean cools down. The onset of convection in the cumulates, that results in heat piping, is identified in Figure S5A by the initial peak in the heat piping curve (red line). In Figure S5B, it corresponds to the reduction in the cooling rate of the magma ocean and in the growth of the plagioclase crust. Heat piping then slowly decreases as the cumulates cool down and less melt is produced. The internal heating diminishes as the magma ocean shrinks, although preferential partitioning of the heat-producing elements in the liquid is taken into account. Radioactive decay of the heat sources plays a minor role in the internal heating, but is still accounted for. The conductive heat flux from the cumulates is minor for most of the crystallization except the end, when the top of the cumulates solidifies following a steep temperature gradient associated with the crystallization of KREEP. This steep gradient results in increased conduction from the cumulates, and the end of the magma ocean crystallization is controlled by the balance between conductive heat fluxes from the cumulates and through the crust.

### 1.3.2. Effect of the various heat fluxes and sources

In Figure S6, for a case with $k_{crust}$ = 2 W/m/K with heat diffusion in the cumulates (i.e. neglecting heat-piping), we show the effects of removing the conductive heat flux at the bottom of the magma ocean, removing internal heating in the magma ocean, removing the effect of latent heat, and changing the crystallization model. The latent heat release upon crystallization has the strongest effect, prolonging by a factor of 2 the duration of the magma ocean solidification when it is included. For a 1000-km-deep magma ocean, including internal heating in the magma ocean delays the solidification by about 20% of the total time. Due to the steep gradient of the crystallization temperature at the top of the cumulates (see Figure S1) reached at the end of magma ocean solidification, the conductive heat flux from the cumulates becomes important towards the end of the solidification. This induces a protracted crystallization of the last few km of the magma ocean, plotting as a long tail in the black lines on Figures S6B and D. Although changing the crystallization model changes the crystallization temperature profile as well as the final thickness of the crust and final temperature, the most prominent difference it induces is in the distribution of heat sources. In the case of a 1000-km-deep magma ocean, a substantial amount of heat sources remains trapped in the unmolten lower mantle and never enters the heat budget of the magma ocean, while for a whole-mantle magma ocean the entire heat-producing elements content of the bulk silicate Moon is partitioned into the magma ocean, resulting in a higher internal heating of the late magma ocean. This can be clearly seen in Figure S6 by comparing the red and dashed black lines corresponding to the two crystallization models, without internal heating. While the two lines are very close to each other, the blue and solid black lines corresponding to the two crystallization models, but with internal heating, depart significantly from one another.

2. Effect of a dense and weak, late crystallizing layer on heat piping

In order to assess the effects of compositional buoyancy of a late crystallized, dense and weak layer as introduced by (*15*) and studied by (*16,17*), we ran tests based on our fiducial value of $k_{crust}$ = 2 W/m/K and two values of the reference viscosity: $10^{19}$ and $10^{21}$ Pa s, along with the set-up described in Materials and Methods section 2.2.3 and Table S1. For $\eta_{ref}$ = $10^{21}$ Pa s, as previously reported by (*16*), no mobilization of the dense and weak layer occurs during the magma ocean solidification, and the time-series are similar to those obtained in the purely thermal case. For $\eta_{ref}$= $10^{19}$ Pa s mobilization does occur, and the return flow associated with the overturn of dense material causes a late surge in the heat-piping flux, as shown in Figure S7. This results in an increase of the total solidification time of ≈ 17% in the thermo-chemical case with respect to the corresponding (2-D) purely thermal case, which translates into a similar range for $t_0$ and a shift of the range for $t_{KREEP}$ of 50 Myr toward younger values with respect to the low end-member reference viscosity case.

3. Sensitivity of the results to the parameters

Figure S8 presents the cases that reached the same fit when time series for $k_{crust}$ = 4 and 1.5 W/m/K for a 1000 km-deep LMO. In the case of a 1000 km-deep LMO, the higher value of $k_{crust}$= leads to a configuration closest to the original two-stage model from 17 and provides the most fitting cases, but is likely significantly underestimating the duration of the magma ocean solidification both because of the use of a high crustal thermal conductivity and the neglect of heat

piping in the cumulates. It is also only marginally compatible with Kal009 data, producing a high enough $\varepsilon^{176}$Hf reservoir only in less than 5% of the cases. The lowest value of $k_{crust}$, also physically unlikely, results in a later formation of KREEP (between 4.13 and 4.25 Ga) but a similar Moon formation time range as for our fiducial case.

The variations in $t_0$ and $t_{KREEP}$ obtained when varying $\eta_{ref}$ and keeping $k_{crust}$ constant (2 W/m/K) are presented in Table S5: $t_0$ is remarably constant (between 4.45 and 4.40 Ga) while $t_{KREEP}$ is more affected by the lifetime of the LMO (the oldest values are obtained for $\eta_{ref}=10^{22}$ Pa s, where the LMO is the shortest because heat-piping is not efficient for lack of convection in the cumulates).

For different initial depths of the LMO, although the shape of the time-series are different (see Fig, S6), the results are very similar to our fiducial case ($t_0$ between 4.40 and 4.44 Ga and $t_{KREEP}$ between 4.20 and 4.29 Ga), as discussed in the main text. The good agreement among cases having a realistic viscosity ($\eta_{ref}\leq 10^{21}$ Pa s) suggests the robustness of these results.

## 4. Formation of a cumulates reservoir compatible with the isotopic signature of lunar meteorite Kal009

For the cases selected by the KREEP data fitting process based on the time series of our fiducial thermal evolution model, we computed the cumulates whole-rock isotopic ratio for the $^{176}$Lu–$^{176}$Hf system (as described in Materials and Methods section 3.2). Figure S9 shows a close-up of the whole-rock $\varepsilon^{176}$Hf radial distribution between radii 1450 km and 1500 km where this value reaches its maximum. The spread of the $\varepsilon^{176}$Hf in this reservoir clearly overlaps with the values measured for the lunar meteorite Kal 009 (12.9±4.620 for an updated age of 3.369 Ga from

(*25*)). We further select the cases that also match $\varepsilon^{176}$Hf measured for Kal 009 (Table S4). This filters the late Moon-forming cases, eventually constraining our estimate for $t_0$ and $t_{KREEP}$ to early values (4.40 to 4.45 Ga and 4.22 to 4.27 Ga respectively), as presented in Figure S9B and Table S5.

5. Hf isotopic composition of the lunar zircon

As discussed in the main text, our estimate of the age of the Moon is at odds with a recent estimate based on lunar zircons, according to which the age of the Moon should be 4.51±0.01 Ga (*23*). One potential problem with the lunar zircon Hf isotopic data is that they must be corrected for the effects of secondary neutron capture during cosmic ray exposure (CRE). CRE-effects on Hf isotopes in lunar samples can be quite significant (*43,44*), and accordingly, (*23*) corrected their zircon data, as well as previously reported data (*45*), for CRE-effects using the correlation of $^{176}$Hf/$^{177}$Hf versus $^{178}$Hf/$^{177}$ Hf observed for whole-rock samples (*43,46*). However, the CRE effects on $^{176}$Hf/$^{177}$Hf depend, in part, on the Lu/Hf ratio of the sample, and so the $^{176}$Hf/$^{177}$Hf–$^{178}$Hf/$^{177}$Hf correlation defined by whole-rock samples cannot easily be applied to zircons, which have much lower Lu/Hf than the whole-rocks. Intriguingly, the least radiogenic Hf isotopic compositions in the dataset from (*23*) have been obtained for zircons from sample 14163, which is known to have very large CRE effects (*47*). As the CRE corrections lower $^{176}$Hf/$^{177}$Hf, it is quite possible that the correction method employed by (*23*) resulted in an overcorrection. Further, the authors also applied CRE corrections to previously published data (*45*) for zircons from sample 14321. For this sample, large variations in the $^{178}$Hf/$^{177}$Hf were observed, which (*23*) attribute to CRE-effects and, consequently, used these data to correct measured $^{176}$Hf/$^{177}$Hf for these effects.

However, bulk rock analyses of sample 14321 show no significant CRE effects on Hf isotopic composition (*44,47*), suggesting that the anomalous $^{178}Hf/^{177}Hf$ for 14321 zircons reported in an earlier study (*45*) are not due to CRE and that, therefore, these zircon data should not be corrected for CRE effects.

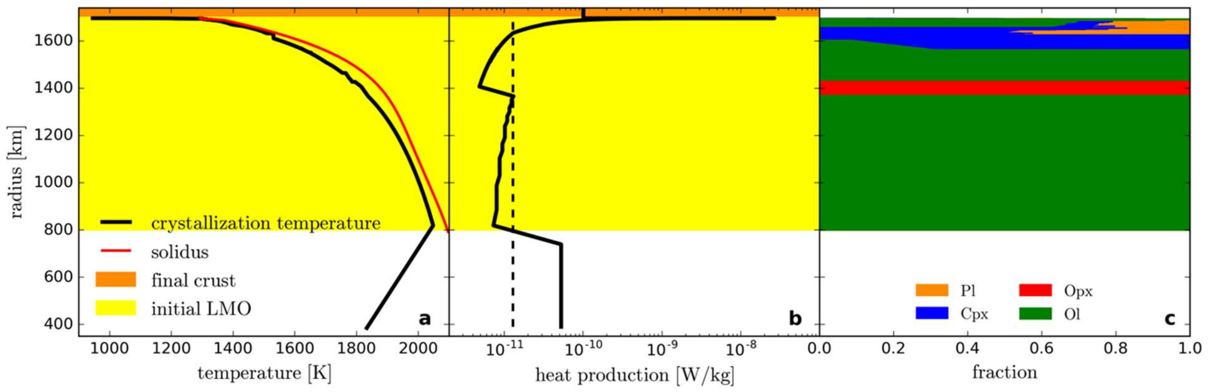


**Fig. S1.**

(**A**) Radial profile of the crystallization temperature (black) and solidus (red) corresponding to our crystallization sequence. The initial depth of the magma ocean is highlighted in yellow and the final depth of the crust is highlighted in orange. (**B**) Radial profile of the specific heat production. The dashed line indicates the average heat production in the mafic cumulates. (**C**) Fraction of four main minerals produced by the crystallization model. For olivine, orthopyroxene, and clinopyroxene, the crystallization position corresponds to the final emplacement in the cumulate pile, plagioclase (orange) is represented where it crystallizes, but then rises to the surface to form the flotation crust. A close-up plot of the crystallization sequence on the shallowest layer with all minerals is shown in Figure S2.

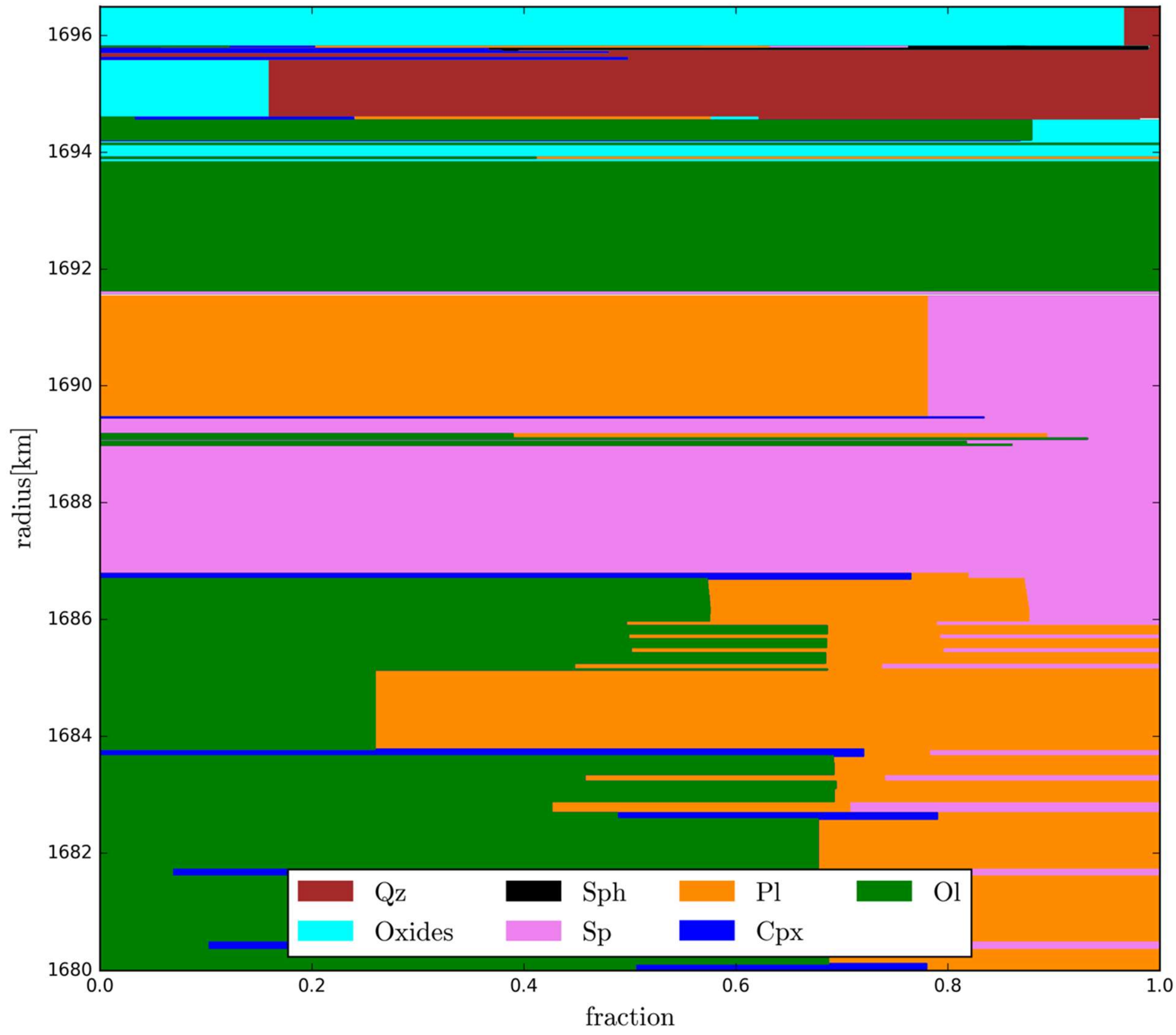


**Fig. S2.**

Close-up of the latest (i.e. shallowest) crystallized layers of the LMO. This region, including KREEP, is enriched in incompatible elements. Oxides include rhombohedral oxides, ortho-oxides and rutile.

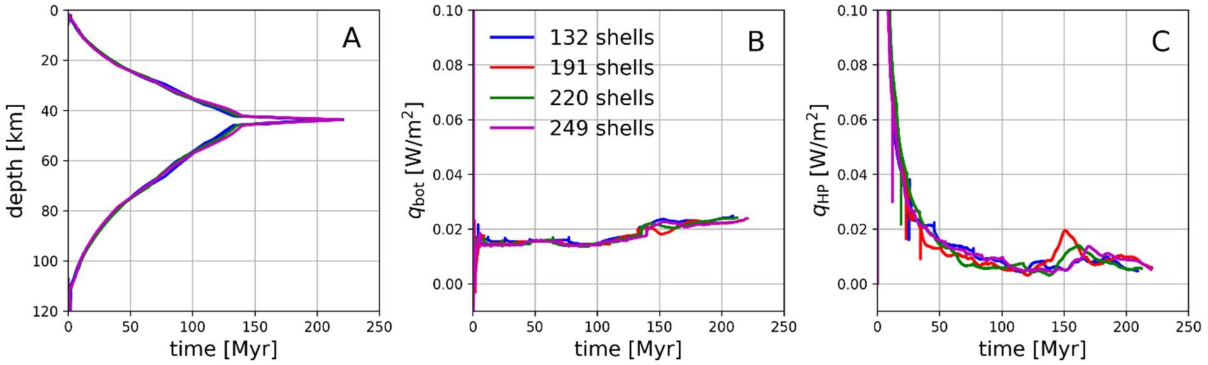


**Fig. S3.**

Convergence tests made on 2-D cylindrical grids for $k_{crust}$ = 2 W/m/K and $\eta_{ref}$ = $10^{19}$ Pa s, with a radial resolution of 132 shells (blue), 191 shells (red), 220 shells (green), and 249 shells (magenta).

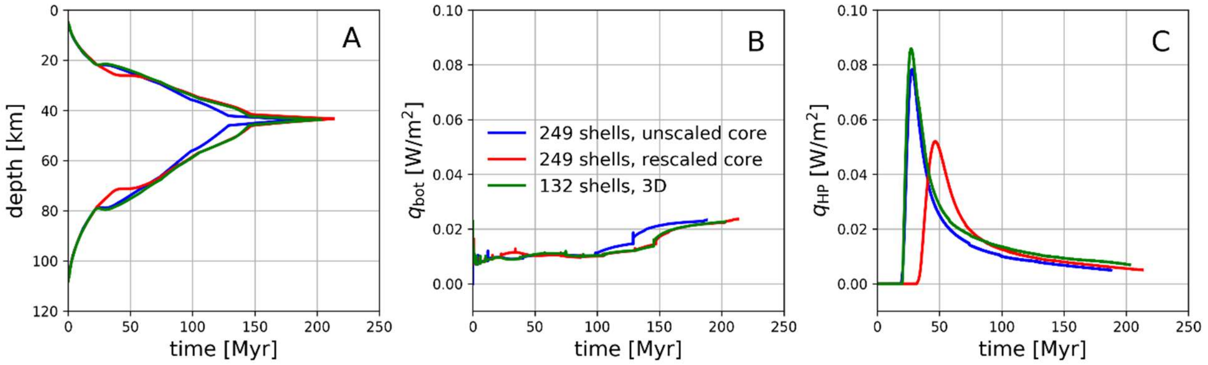


**Fig. S4.**

Comparison between a 3-D simulation with 132 radial shells (green), a 2-D simulation with 249 radial shells with the actual lunar core radius (blue), and a 2-D simulation with 249 radial shells but a core radius rescaled according to ref. (*39*) (red). As in Figure S3, panel (**A**) represents the evolution of the bottom of the crust and magma ocean, panel (**B**) that of the conductive heat flux at the top of the cumulates, and panel (**C**) that of the heat-piping flux.

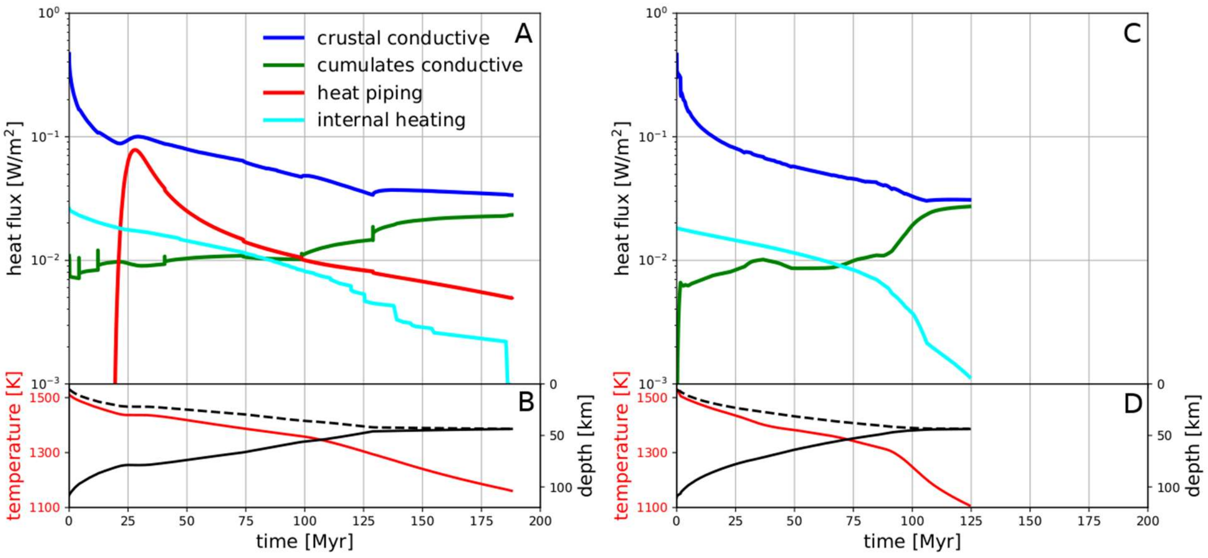


**Fig. S5.**

(**A**) Time evolution of the heat fluxes and sources for the fiducial case accounting for heat piping with $k_{crust}$ = 2 W/m/K, $\eta_{ref}$ = $10^{21}$ Pa s, and (**B**) corresponding evolution of the temperature (red) and magma ocean's top and bottom boundaries (black). (**C**) Time evolution of the heat fluxes and sources for the reference, purely diffusive case, i.e. with $k_{crust}$= 2 W/m/K but neglecting convection and heat piping, and (**D**) corresponding evolution of the temperature (red) and magma ocean's top and bottom boundaries (black).

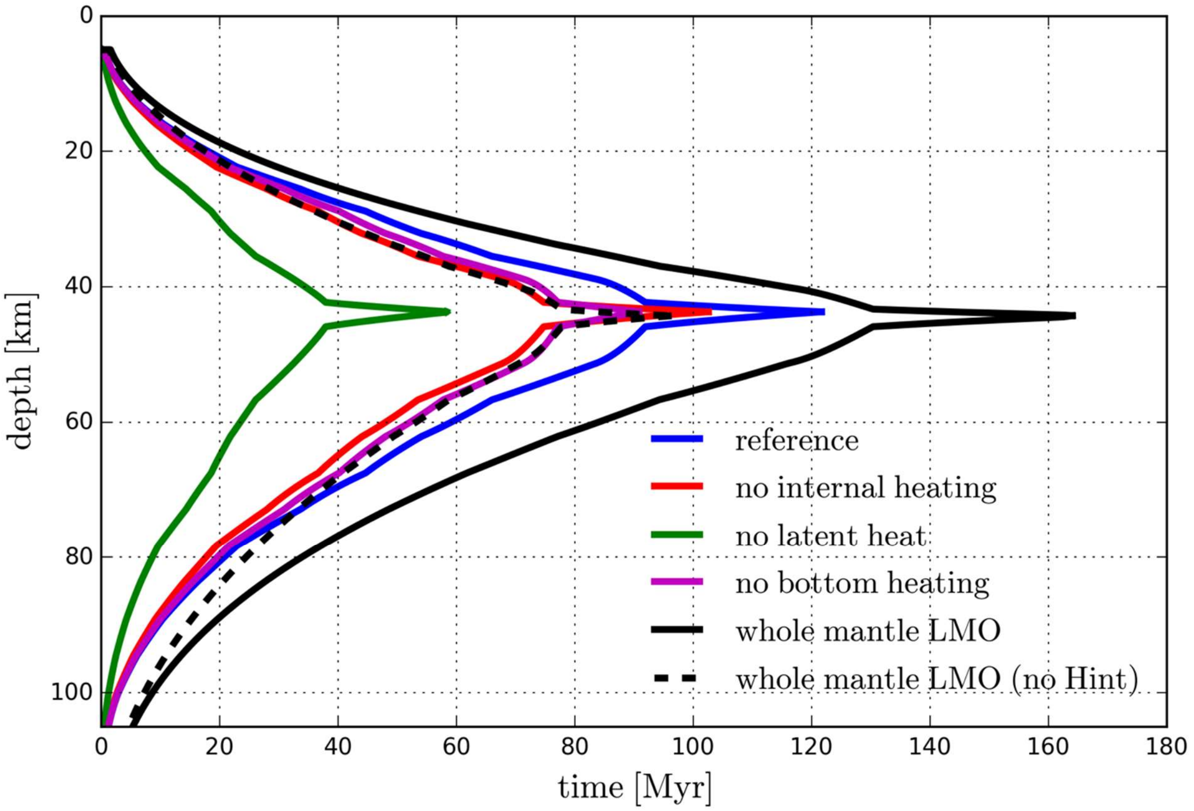


**Fig. S6.**

Evolution of the magma ocean's top and bottom depths for the reference, purely diffusive model with $k_{crust}$= 2 W/m/K but neglecting convection and heat piping in blue, the same model without internal heating (red), without conductive bottom heating (purple), without latent heat effect (green), for the case of an initially 1350 km-deep magma ocean with (solid black) and without (dashed black) internal heating.

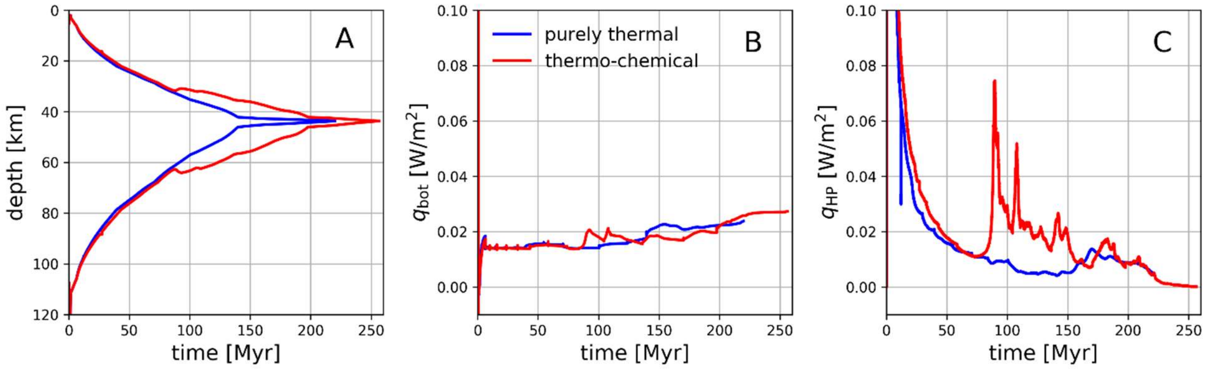


**Fig. S7.**

Comparison between the time-series of the purely thermal (blue curves) and the thermo-chemical case (red curves) with $\eta_{ref}$ = $10^{19}$ Pa s, for the depths of the bottom of the crust and the magma ocean (**A**), the conductive heat flux from the solid cumulates (**B**) and the heat-piping flux (**C**). In the thermo-chemical case, the viscosity in the dense layer is lowered by one order of magnitude, as per equation [11]. Both cases are ran on 2-D grids.

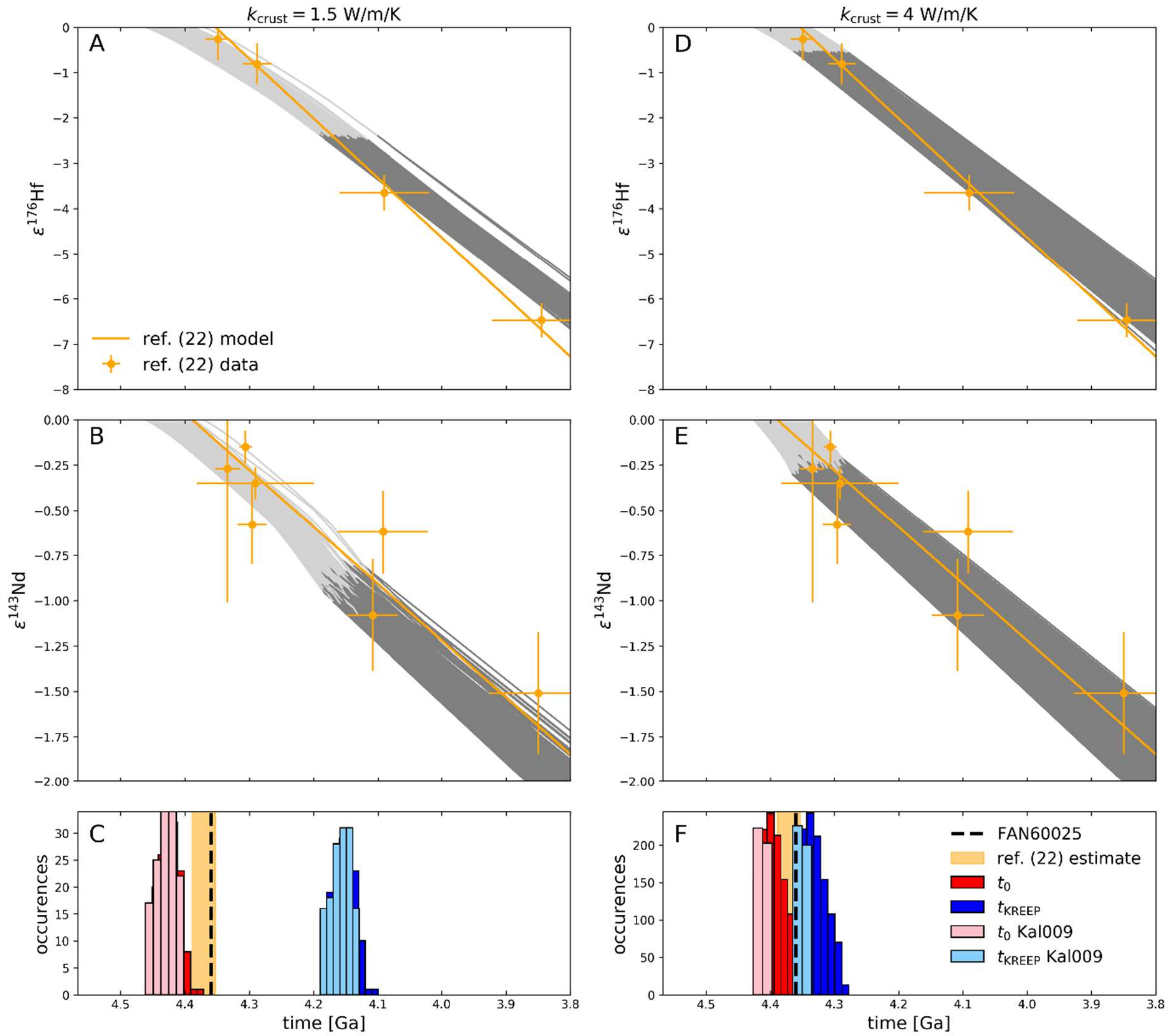


**Fig. S8.**

Results of the Monte-Carlo fractionation simulations using time-series based on a 1000 km-deep LMO with $\eta_{ref}$ = $10^{21}$ Pa s and $k_{crust}$ = 1.5 (**A**-**C**), and 4 W/m/K (**D**-**F**). The free parameters are the values of the partition coefficients and the time of the Moon-forming impact ($t_0$).

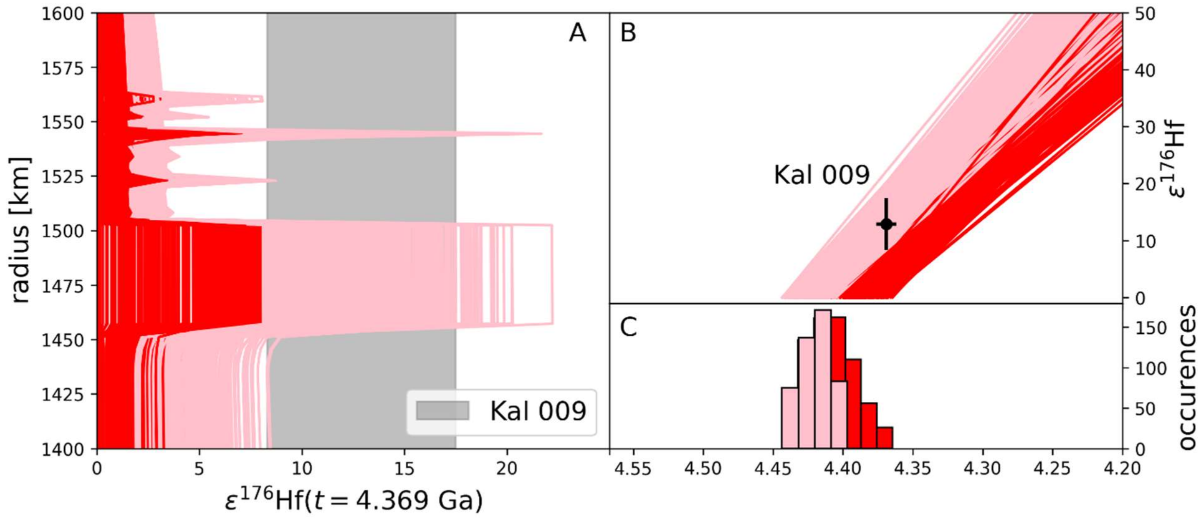


**Fig. S9.**

(**A**) Radial distribution of $\varepsilon^{176}$Hf in the solid cumulate pile at 4.369 Ga (age estimated for Kal 009 (*24*)) for all cases matching KREEP isotopic ratios (red and pink lines). The initial $\varepsilon^{176}$Hf value for Kal 009 (*25*) corresponds to the grey shaded area (within standard deviations). (**B**) $\varepsilon^{176}$Hf evolution of the highly fractionated reservoir of all cases selected by the KREEP fitting process. Those cases that also cross the uncertainty ellipsoid of Kal 009 (black point and error bars) are plotted in pink, while those which do not are plotted in red (as in Figure 4). (**C**) $t_0$ distribution, as plotted in Figure 4 of the main text.

| Quantity | Value | Unit | Quantity | Value | Unit |
|---|---|---|---|---|---|
| **Core** | | | **Magma ocean** | | |
| $c_{p,\mathrm{core}}$ | 840 | J/kg/K | $h_{\mathrm{MO}}$ | variable | W/kg |
| $\rho_{\mathrm{core}}$ | 7200 | kg/m$^3$ | $f$ | 0.39 | |
| $R_{\mathrm{core}}$ | 390 | km | **Crust** | | |
| **Cumulates** | | | $k_{\mathrm{crust}}$ | 1.5 to 4 | W/m/K |
| $h_0$ | $1.43 \times 10^{-11}$ | W/kg | $\kappa_{\mathrm{crust}}$ | $4.6 \times 10^{-7}$ to $1.2 \times 10^{-6}$ | m$^2$/s |
| $\eta_{\mathrm{ref}}$ | $10^{19}$ to $10^{22}$ | Pa s | $\rho_{\mathrm{crust}}$ | 2715 | kg/m$^3$ |
| $E^*$ | 335 | kJ/mol | $h_{\mathrm{crust},0}$ | $1.01 \times 10^{-10}$ | W/kg |
| $V^*$ | $4 \times 10^{-6}$ | m$^3$/mol | **Reference** | | |
| $R$ | 8.314 | J/mol/K | $D_{\mathrm{ref}}$ | 1350 | km |
| $p_{\mathrm{ref}}$ | 3 | GPa | $\Delta T$ | 1930 | K |
| $T_{\mathrm{ref}}$ | 1600 | K | $T_s$ | 250 | K |
| **Dense weak layer** | | | $c_p$ | 1200 | J/kg/K |
| $\rho_1$ | 3775 | kg/m$^3$ | $\alpha$ | $3.0 \times 10^{-5}$ | 1/K |
| $h_1$ | $1.27 \times 10^{10}$ | W/kg | $\rho$ | 3245 | kg/m$^3$ |
| $D_1$ | 27 | km | $k$ | 4.0 | W/m/K |
| $\Delta\eta$ | 10 | - | $\kappa$ | $10^{-6}$ | m$^2$/s |
| | | | $L$ | $5 \times 10^5$ | J/kg |

**Table S1.**

Physical quantities used. Values computed from our crystallization model are highlighted in blue and model parameters in red.

| | | | |
|---|---|---|---|
| $^{176}Lu$-$^{176}Hf$ | | $^{147}Sm$-$^{143}Nd$ | |
| Half-life | 53.56 Gyr[a] | Half-life | 152.91 Gyr[a] |
| **chondrites** | | | |
| $^{176}Lu$ / $^{174}Hf$ | 0.0336[b] | $^{147}Sm$ / $^{144}Nd$ | 0.1960[b] |
| $^{176}Hf$ / $^{174}Hf$ | 0.282785[b] | $^{143}Nd$ / $^{144}Nd$ | 0.512630[b] |
| **KREEP** | | | |
| $^{176}Lu$ / $^{174}Hf$ | 0.0153 ± 0.0033[a] | $^{147}Sm$ / $^{144}Nd$ | 0.1723 ± 0.0019[a] |

**Table S2.**

Half-lives and present-day chondritic values of the parent-to-stable daughter and radiogenic-to-stable daughter ratios for the two isotopic systems used. references: [a]: (*22*), [b]: (*40*)

| Mineral | $K_{Lu}$ | $K_{Hf}$ | $K_{Sm}$ | $K_{Nd}$ |
|---|---|---|---|---|
| Olivine | 0.0089±11[a] | 0.0008±1[a] | 0.00032±4[a] | 0.00008±1[a] |
| Clinopyroxene | 0.74±12[a] | 0.37±10[a] | 0.459±78[a] | 0.273±51[a] |
| Orthopyroxene | 0.181±31[a] | 0.047±17[a] | 0.0251±3[a] | 0.0120±2[a] |
| Plagioclase | 0.092±4[b] | 0.1483±25[b] | 0.1650±11[b] | 0.203±7[b] |
| Ilmenite | 0.0545±76[c] | 0.52±4[c] | 0.0037±7[c] | 0.0055±4[c] |
| Spinel | 0.28±9[c] | 0.65±17[c] | 0.007±4[c] | 0.0037±21[c] |
| Quartz | 0.01424±104[b] | 0.03025±75[b] | 0.0140±2[b] | 0.016±2[b] |
| Whitlockite | 1.902±57[d] | 0.01216±90[d] | 12.67±51[d] | 10.18±26[d] |
| Sphene | 34.1±7[e] | 27.90±1.49[e] | 6.23±25[e] | 14.01±32[e] |

**Table S3.**
Partition coefficients. References: [a] (*48*), [b] (*49*), [c] (*50*), [d] (*51*), [e] (*52*).

| **Name** | **Age [Ma]** | **$\varepsilon^{176}$Hf** | **$\varepsilon^{143}$Nd** |
|---|---|---|---|
| 15386 | 3845±77[a] | -6.47±0.38[a] | -1.51±0.335[b] |
| 72275,383 | 4090±70[a] | -3.65±0.39[a] | -0.62±0.63[c] |
| NWA773 | 2993±32[d] | unknown | -4.5±0.3[d] |
| 77215 | 4288±22[e] | -0.81±45[a] | -0.54±0.09[e] |
| 78238 | 4349±19[e] | -0.26±0.48[a] | -0.27±0.74[e] |
| 14304,267 | 4108±40[e] | unknown | -1.08±0.23[e] |
| 15455,247 | 4291±91[e] | unknown | -0.35±0.31[e] |
| 76535 | 4306±10[e] | unknown | -0.15±0.22[e] |
| Kal009 | 4369±7[f] | +12±4.6[g] | unknown |

**Table S4.**

Ages and εX values of the KREEP samples used to select the fractionation models. The first three samples are KREEP basalts and the five last are Mg-suite rocks. The last line corresponds to the basalt sample Kal 009 used to refine the fit. References: [a] *(22)*, [b] (*53*), [c] (*54*), [d] (*55*), [e] (*9*), [f] (*24*), [g] (*25*)

| $\eta_{ref}$ [Pa s] | $t_0$ [Ga] | $T_{KREEP}$ [Ga] | $t_0$ [Ga] (fitted to Kal 009) | $T_{KREEP}$ [Ga] (fitted to Kal 009) |
|---|---|---|---|---|
| $10^{22}$ | 4.44-4.35 | 4.32-4.23 | 4.44-4.40 | 4.32-4.28 |
| $10^{21}$ | 4.44-4.36 | 4.27-4.19 | 4.44-4.40 | 4.27-4.22 |
| $10^{20}$ | 4.44-4.36 | 4.29-4.20 | 4.45-4.40 | 4.29-4.24 |
| $10^{19}$ | 4.45-4.36 | 4.26-4.18 | 4.45-4.40 | 4.26-4.21 |

**Table S5.**

Moon formation time ($t_0$) and KREEP isotlation time ($t_{KREEP}$) spans for all values of the reference viscosity investigated, with our fiducial value of the crustal thermal conductivity: $k_{crust}$ = 2 W/m/K.

## REFERENCES AND NOTES

1. A. C. Barr, On the origin of Earth's Moon. *J. Geophys. Res. Planets* **121**, 1573–1601 (2016).

2. L. T. Elkins-Tanton, S. Burgess, Q.-Z. Yin, The lunar magma ocean: Reconciling the solidification process with lunar petrology and geochronology. *Earth Planet. Sci. Lett.* **304**, 326–336 (2011).

3. V. Perera, A. P. Jackson, L. T. Elkins-Tanton, E. Asphaug, Effect of reimpacting debris on the solidification of the lunar magma ocean. *J. Geophys. Res. Planets* **123**, 1168–1191 (2018).

4. J. A. Wood, J. S. Dickey Jr., U. B. Marvin, B. N. Powell, Lunar anorthosites and geophysical model of the Moon, in *Proceedings of the Apollo 11 Lunar Science Conference* (Pergammon Press, 1970), pp. 897–925.

5. J. Meyer, L. Elkins-Tanton, J. Wisdom, Coupled thermal-orbital evolution of the early Moon. *Icarus* **208**, 1–10 (2010).

6. Z. Tian, J. Wisdom, L. Elkins-Tanton, Coupled thermal-orbital evolution of the early Earth-Moon system with a fast-spinning Earth. *Icarus* **281**, 90–102 (2017).

7. E. M. A. Chen, F. Nimmo, Tidal dissipation in the lunar magma ocean and its effect on the early evolution of the Earth-Moon system. *Icarus* **275**, 132–142 (2016).

8. V. Perera, A. P. Jackson, L. T. Elkins-Tanton, E. Asphaug, Effect of reimpacting debris on the solidification of the lunar magma ocean. *J. Geophys. Res. Planets* **123**, 1168–1191 (2018).

9. M. A. Wieczorek, B. L. Joliff, A. Khan, M. E. Pritchard, B. P. Weiss, J. G. Williams, L. L. Hood, K. Righter, C. R. Neal, C. K. Shearer, I. S. McCallum, S. Tompkins, B. R. Hawke, C. Peterson, J. J. Gillis, B. Bussey, The constitution and structure of the lunar interior. *Rev. Mineral. Geochem.* **60**, 221–364 (2006).

10. J. M. Barnlund, A. M. Hofmeister, Heat transfer in plagioclase feldspar. *Am. Mineral.* **97**, 1145–1154 (2012).

11. M. Maurice, N. Tosi, H. Samuel, A.-C. Plesa, D. Breuer, C. Hüttig, Onset of solid-state mantle convection and mixing during magma ocean solidification. *J. Geophys. Res. Planets* **122**, 577–598 (2017).

12. C.-E. Boukaré, E. M. Parmentier, S. W. Parman, Timing of mantle overturn during magma ocean solidification. *Earth Planet. Sci. Lett.* **491**, 216–225 (2018).

13. A. Morison, S. Labrosse, R. Deguen, T. Alboussière, Timescale of overturn in a magma ocean cumulate. *Earth Planet. Sci. Lett.* **516**, 25–36 (2019).

14. G. Hirth, D. Kohlstedt, Rheology of the upper mantle and the mantle wedge: A view from the experimentalists, in *Inside the Subduction Factory*, J. Eiler, Ed. (Geophysical Monograph Series, AGU, 2003), vol. 138, pp. 83–105.

15. P. C. Hess, E. M. Parmentier, A model for the thermal and chemical evolution of the Moon's interior: Implication for the onset of mare volcanism. *Earth Planet. Sci. Lett.* **134**, 501–514 (1995).

16. S. Yu, N. Tosi, S. Schwinger, M. Maurice, D. Breuer, L. Xiao, Overturn of ilmenite-bearing cumulates in a rheologically weak lunar mantle. *J. Geophys. Res. Planets* **124** 418–436 (2019).

17. Y. Zhao, J. de Vries, A. P. van den Berg, M. H G. Jacobs, W. van Westrenen, The participation of ilmenite-bearing cumulates in lunar mantle. *Earth Planet. Sci. Lett.* **511,** 1–11 (2019).

18. C. K. Shearer, P. C. Hess, M. A. Wieczorek, M. E. Pritchard, E. M. Parmentier, L. E. Borg, J. Longhi, L T. Elkins-Tanton, C. R. Neal, I. Antonenko, R. N. Canup, A. N. Halliday, T. L. Grove, B. H. Hager, D.-C. Lee, U. Wiechert, Thermal and magmatic evolution of the Moon. *Rev. Mineral. Geochem.* **60**, 365–518 (2006).

19. J. de Vries, A. van den Berg, W. van Westrenen, Formation and evolution of a lunar core from ilmenite-rich magma ocean cumulates. *Earth Planet. Sci. Lett.* **292**, 139–147 (2010).

20. L. E. Borg, A. M. Gaffney, C. K. Shearer, A review of lunar chronology revealing a preponderance of 4.34-4.37 Ga ages. *Meteorit. Planet. Sci.* **50**, 715–732 (2015).

21. L. E. Borg, J. N. Connelly, M. Boyet, R. W. Carlson, Chronological evidence that the Moon is either young or did not have a global magma ocean. *Nature* **477**, 70–72 (2011).

22. A. M. Gaffney, L. E. Borg, A young solidification age for the lunar magma ocean. *Geochim. Cosmochim. Acta* **140**, 227–240 (2014).

23. M. Barboni, P. Boehnke, B. Keller, I. E. Kohl, B. Schoene, E. D. Young, K. D. McKeegan, Early formation of the Moon 4.51 bilion years ago. *Sci. Adv.* **3**, e1602365 (2017).

24. J. F. Snape, N. M. Curran, M. J. Whitehouse, A. A. Nemchin, K. H. Joy, T. Hopkinson, M. Anand, J. J. Belluci, G. G. Kenny, Ancient volcanism on the Moon: Insights from Pb isotopes in the MIL 13317 and Kalahari 009 lunar meteorites. *Earth Planet. Sci. Lett.* **502**, 84–95 (2018).

25. A. K. Sokol, V. A. Fernandes, T. Schultz, A. Bischoff, R. Burgess, R. N. Clayton, K. Münker, K. Nishiizumi, H. Palme, L. Schultz, G. Weckwerth, K. Mezger, M. Horstmann, Geochemistry, petrology and ages of the lunar meteorites Kalahari 008 and 009: New constraints on early evolution. *Geochim. Cosmochim. Acta* **72**, 4845–4873 (2008).

26. F. Albarède, Volatile accretion history of the terrestrial planets and dynamic implications. *Nature* **461**, 1227–1233 (2009).

27. J. F. Rudge, T. Kleine, B. Bourdon, Broad bounds on Earth's accretion and core formation constrained by geochemical models. *Nat. Geosci.* **3**, 439–443 (2010).

28. C. J. Allègre, G. Manhès, C. Göpel, The age of the Earth. *Geochim. Cosmochim. Acta* **59**, 1445–1456 (1995).

29. B. J. Wood, A. N. Halliday, The lead isotopic age of the Earth can be explained by core formation alone. *Nature* **465**, 767–770 (2010).

30. S. A. Jacobson, A. Morbidelli, S. N. Raymond, D. P. O'Brien, K. J. Walsh, D. C. Rubie, Highly siderophile elements in Earth's mantle as a clock for the Moon-forming impact. *Nature* **508**, 84–87 (2014).

31. W. F. Bottke, D. Vokrouchlický, S. Marchi, T. Swindle, E. R. D. Scott, J. R. Weirich, H. Levison, Lunar formation. Dating the Moon-forming impact event with asteroidal meteorites. *Science* **348**, 321–323 (2015).

32. J. D. Davenport, C. R. Neal, G A. Snyder, D. Bolster, J. Longhi, Forward, reverse and FXMOTR modelling of the LMO: A new look at the bulk composition of the LMO, in *45th Lunar Planetary Science Conference* (LPI press, 2014).

33. J. F. Rapp, D. S. Draper, Fractional crystallization of the lunar magma ocean: Updating the dominant paradigm. *Meteor. Planet. Sci.* **53,** 1432–1455 (2018).

34. P. M. Smith, P. D. Asimov, Adiabat_1ph: A new public front-end to the MELTS, pMELTS, and pHMELTS models. *Geochem. Geophys. Geosyst.* **6**, Q02004 (2005).

35. S. Schwinger, D. Breuer, Modeling the thermochemical evolution of the lunar magma ocean using igneous crystallization programs, in *AGU Fall Meeting* (AGU press, 2018).

36. H. St. C. O'Neill, The origin of the moon and the early history of the earth—A chemical model. Part 1: The moon. *Gechim. Cosmochim. Acta* **55**, 1135–1157 (1991).

37. S. R. Taylor, *Planetary Science, A Lunar Perspective* (Lunar and Planetary Institute, 1982).

38. M. A. Wieczorek, G. A. Neumann, F. Nimmo, W. S. Kiefer, G. J. Taylor, H. J. Melosh, R. J. Phillips, S. C. Solomon, J. C. Andrews-Hanna, S. W. Asmar, A. S. Konopliv, F. G. Lemoine, D. E. Smith, M. M. Watkins, J. G. Wiliams, M. T. Zuber, The crust of the Moon as seen by GRAIL. *Science* **339**, 671–675 (2013).

39. A.-C. Plesa, N. Tosi, C. Hüttig, Thermo-chemical convection in planetary mantles: Advection methods and magma ocean overturn simulations, in *Integrated Information and Computing Systems for Natural, Spatial and Social Sciences* (IGI Global, 2012), pp. 302–323.

40. A. Bouvier, J. D. Vervoort, J. P. Patchett, The Lu-Hf and Sm-Nd isotopic composition of CHUR: Constraints from unequilibrated chondrites and implicatoins for the bulk composition of terrestrial planets. *Earth Planet. Sci. Lett.* **273**, 48–57 (2008).

41. J. W. Hernlund, P. J. Tackley, Modelling mantle convection in the spherical annulus. *Phys. Earth Planet. Inter.* **171**, 48–54 (2008).

42. P. van Keken, Cylindrical scaling for dynamical cooling models of the Earth. *Phys. Earth Planet. Inter.* **124**, 119–130 (2001).

43. T. S. Kruijer, T. Kleine, Tungsten isotopes and the origin of the Moon. *Earth Planet. Sci. Lett.* **475**, 15–24 (2017).

44. P. Sprung, T. Kleine, E. E. Scherer, Isotopic evidence for chondritic Lu/Hf and Sm/Nd of the Moon. *Earth Planet. Sci. Lett.* **380**, 77–87 (2013).

45. D. J. Taylor, K. D. McKeegan, T. M. Harrison, Lu-Hf zirconevidence for rapid lunar differentiation *Earth Planet. Sci. Lett.* **279**, 157–164 (2009).

46. P. Sprung, E. E. Scherer, D. Upadhyay, I. Leya, K. Mezger, Non-nucleosynthetic heterogeneity in non-radiogenic stable Hf isotopes: Implications for early solar system chronology. *Earth Planet. Sci. Lett.* **295**, 1–11 (2010).

47. T. Kruijer, T. Kleine, M. Fischer-Gödde, P. Sprung, Lunar tungsten isotopic evidence for the late veneer. *Nature* **520**, 534–537 (2015).

48. P. McDade, J. D. Blundy, B. J. Wood, Trace elements partitioning on the Tinaquilo lherzolite solidus at 15 GPa. *Phys. Earth Planet. Inter.* **139,** 129–147 (2003).

49. W. P. Nash, H. R. Crecraft, Partition coefficients for trace elements in silicic magmas. *Geochim. Cosmochim. Acta* **49**, 2309–2322 (1985).

50. S. Klemme, D. Günther, S. Prowatke, T. Zack, The partitioning of trace elements between ilmenite, ulvospinel, armalcolite and silicate melts with implications for the early differentiation of the Moon. *Chem. Geol.* **234**, 251–263 (2006).

51. S. Prowatke, S. Klemme, Trace elements partitioning between apatite and silicate melts. *Geochim. Cosmochim. Acta* **70**, 4513–4527 (2006).

52. S. Prowatke, S. Klemme, Effect of melt composition on the partitioning of trace elements between titanite and silicate melt. *Geochim. Cosmochim. Acta* **69**, 695–709 (2005).

53. R. W. Carlson, G. W. Lugmair, Sm–Nd constraints on early lunar differentiation and the evolution of KREEP. *Earth Planet. Sci. Lett.* **45**, 123–132 (1979).

54. C.-Y. Shih, L. E. Nyquist, B. M. Bansal, H. Wiesman, Rb–Sr and Sm–Nd chronology of an Apollo 17 KREEP basalt. *Earth Planet. Sci. Lett.* **108**, 203–215 (1992).

55. L. E. Nyquist, C.-Y. Shih, Y. D. Reese, A. J. Irving, Sm–Nd and Rb–Sr ages for North West Africa 2977, a young lunar gabbro from the PKT, in *72nd Annual Meteoritical Society Meeting* (MetSoc, 2009).
